# Nanoplatforms of Pegylated Gold Nanorods Loaded with Non-Steroidal Anti-Inflammatory Drugs: Delivery and Biological Evaluation

**DOI:** 10.3390/molecules31183328

**Published:** 2026-09-19

**Authors:** Eleni Zygouri, Chrisavgi Gourdoupi, Spyros Kremmydas, Nikos Karamanos, Georgios Malis, George Psomas, Vlasoula Bekiari, Vassilis Tangoulis

**Affiliations:** 1 Laboratory of Inorganic Chemistry, Department of Chemistry, University of Patras, 26504 Patras, Greece; eleni0503zig@hotmail.com; 2 Biochemistry, Biochemical Analysis & Matrix Pathobiology Research Group, Laboratory of Biochemistry, Department of Chemistry, University of Patras, 26504 Patras, Greece; up1061190@ac.upatras.gr (C.G.);; 3 Department of General and Inorganic Chemistry, Faculty of Chemistry, Aristotle University of Thessaloniki, 54124 Thessaloniki, Greece; georgosmalis@gmail.com; 4 Department of Agriculture, University of Patras, 30200 Messolonghi, Greece; bbekiari@upatras.gr

**Keywords:** gold nanorods, PEGylation, fenamic acid, NSAIDs, drug delivery, pH-dependent release, DNA binding, serum albumin, breast cancer, 3D spheroids

## Abstract

This study explored the potential of gold nanorods (AuNRs) functionalized with polyethylene glycol (PEG) and loaded with non-steroidal anti-inflammatory drugs (NSAIDs) fenamic acid (fenH) or its bromo-derivative (4′BrfenH) for targeted drug delivery and preclinical cancer research, given their pH-dependent drug-release profiles and in vitro cytotoxicity in breast cancer cell models. Both formulations demonstrated strong π–π stacking interactions with calf-thymus DNA, suggesting intercalation, and exhibited significant binding affinity to bovine and human serum albumins. Drug release was markedly higher at alkaline pH. As the fenamates are weak acids that are practically insoluble in their neutral form, this pH dependence is attributed principally to the ionization and solubility of the released drug rather than to a responsive carrier. Cytotoxicity assays revealed that AuNRs@PEG@4′BrfenH significantly reduced cell viability in both aggressive (MDA-MB-231) and less metastatic (MCF-7) breast cancer cell lines, particularly at higher concentrations, and caused structural disorganization in 3D spheroid cultures. These findings highlight the promise of AuNRs@PEG@4′BrfenH as a nanoplatform showing pH-dependent release and significant in vitro anticancer activity in breast cancer cell lines and spheroids, supporting its further investigation in relevant tumor models.

## 1. Introduction

Stimuli-responsive gold nanorods (AuNRs) have emerged as highly versatile platforms for targeted drug delivery, offering the potential for controlled and precise release of therapeutic agents. Their unique optical and physicochemical properties, including tunable plasmonic behavior and high surface area, enable the integration of multiple functionalities such as targeting ligands, imaging agents, and therapeutic payloads within a single nanostructure. This multifunctionality allows for enhanced treatment effectiveness and the minimization of side effects, as drug release can be actively triggered by specific biological cues or external stimuli, thereby improving the precision of drug delivery systems [1,2,3,4,5,6,7,8].

Stimuli-responsive nanocarriers [9,10,11,12] are engineered to release their therapeutic payloads in response to specific environmental triggers. These triggers can be classified as endogenous (internal) or exogenous (external). The endogenous stimuli include changes in pH, redox potential, enzymatic activity, and other biochemical signals present in the tumor microenvironment or within diseased tissues [13,14,15,16]. It has been reported that the acidic pH or the presence of specific enzymes such as cathepsins in tumors can be exploited to trigger the release of drugs from AuNRs that are coated with pH-sensitive polymers or enzyme-cleavable linkers [17,18]. On the other hand, the exogenous stimuli involve the application of external physical factors such as light (especially near-infrared, NIR), temperature, magnetic fields, or ultrasound. AuNRs are particularly well-suited for light-triggered drug release due to their strong absorption in the NIR region, which allows for localized heating and subsequent release of the loaded drug at the target site [19]. The ability to combine both endogenous and exogenous triggers in dual- or multi-stimuli-responsive systems further enhances the specificity and control of drug release, reducing off-target effects and improving therapeutic outcomes [19,20].

Gold nanorods can be loaded with a wide variety of therapeutic agents, ranging from small-molecule drugs to large biomolecules. The loading strategies are diverse and depend on the nature of the drug and the intended release mechanism. Small-molecule drugs such as doxorubicin, 5-fluorouracil, resveratrol, chlorambucil, and naproxen have been successfully conjugated to gold nanorods [21,22,23,24,25]. These drugs can be attached via covalent or noncovalent interactions, including ionic or hydrophobic interactions, or through specific binding to surface-modified nanorods. For instance, doxorubicin-conjugated gold nanoparticles have shown increased cell growth inhibition compared to the free drug, and 5-fluorouracil-loaded gold nanoparticles have demonstrated enhanced cytotoxicity in cancer cell lines [21,22,23,24,25].

Biomacromolecules including proteins, peptides, plasmid DNAs, and siRNAs can also be loaded onto gold nanorods. The high surface area and functional flexibility of AuNRs facilitate the attachment of these larger molecules, either through covalent bonding or via surface modifications that enable noncovalent interactions [26]. Finally, targeting ligands and imaging agents can be co-loaded or conjugated to AuNRs, enabling active targeting of specific cell types and facilitating imaging-guided therapy [19,20,21,22,23,24,25,26].

The release of these payloads can be finely controlled by engineering the surface chemistry of the nanorods. For example, drugs can be loaded onto pH-sensitive coatings or linked via bonds that are cleaved by tumor-associated enzymes, ensuring that the therapeutic agents are released only in the desired microenvironment [27,28]. Additionally, the use of NIR light to trigger drug release from AuNR-containing nanofibers or hydrogels allows for on-demand, spatially and temporally controlled delivery, which is particularly advantageous in cancer therapy [29].

Stimuli-responsive AuNRs have been applied in various therapeutic contexts, including (a) cancer therapy, where they enable targeted delivery and controlled release of chemotherapeutic agents, reducing systemic toxicity and enhancing efficacy [19,20]; (b) photothermal therapy (PTT), where AuNRs convert NIR light into heat, enabling the ablation of tumor cells and simultaneous drug release for synergistic effects [30,31]; (c) gene-targeted therapy, where AuNRs serve as carriers for nucleic acids, facilitating their delivery and release in response to specific stimuli [32] and (d) antibacterial applications, where the inherent properties of AuNRs can be harnessed to prevent infections, such as postoperative intraocular infections [33]. The integration of diagnostic and therapeutic functions within a single AuNR platform further expands their utility, allowing for imaging-guided surgery and real-time monitoring of drug delivery [19].

A critical aspect of the clinical translation of gold nanorod-based drug delivery systems is their biocompatibility and stability in physiological environments. PEGylation, the process of modifying the surface of nanoparticles with polyethylene glycol (PEG), is a widely adopted strategy to address several challenges. PEGylation imparts a hydrophilic, inert, and biocompatible “stealth” coating to AuNRs, reducing their aggregation and prolonging their circulation time in the bloodstream by minimizing recognition and clearance by the mononuclear phagocyte system [34,35]. Furthermore, the PEG coating decreases protein adsorption (opsonization) and subsequent immune activation, thereby lowering the risk of adverse immune responses and improving the safety profile of GNRs for in vivo applications [35]. While PEGylation enhances biocompatibility and circulation time, it may also reduce cellular uptake due to the stealth effect. This trade-off must be carefully considered in the design of GNR-based drug delivery systems, balancing the need for prolonged circulation with efficient delivery to target cells [35]. Finally, PEG is an FDA-approved, generally regarded as safe (GRAS) polymer that can be functionalized with various groups for further surface modification, enabling the attachment of targeting ligands, imaging agents, or additional therapeutic molecules [36].

**Figure 1:**
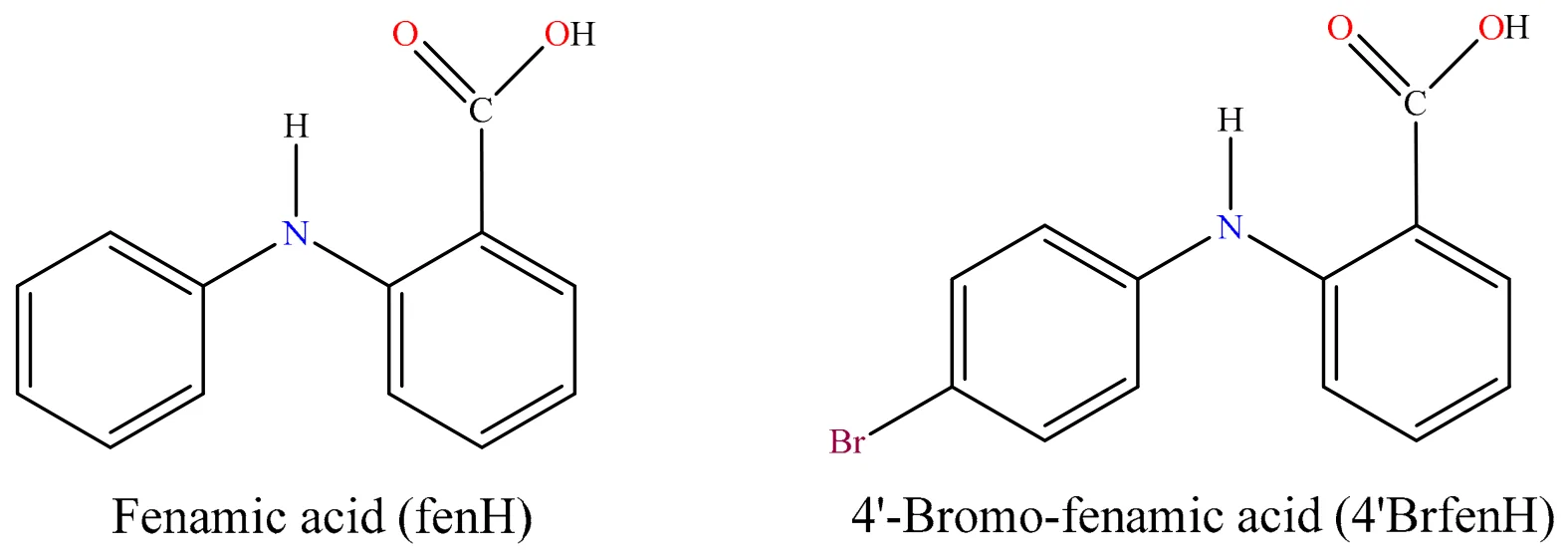
The syntax formulas of fenamic acid (fenH) and 4′-bromofenamic acid (4′BrfenH).

In this study, pegylated AuNRs (AuNRs@PEG) have been developed for targeted delivery of non-steroidal anti-inflammatory drugs (NSAIDs) fenamic acid (fenH) and its bromo-derivative 4′-bromofenamic acid (4′BrfenH) (Figure 1). The evaluation of the potential biological targets of AuNRs@PEG@fenH and AuNRs@PEG@4′BrfenH focused on: (i) their interaction with calf-thymus (CT) DNA studied with UV-vis spectroscopy and DNA-viscosity measurements and via their ability to displace the reference intercalation compound ethidium bromide (EB) from its DNA-adduct, explored with fluorescence emission spectroscopy, and (ii) their affinity for human serum albumin (HSA) and bovine serum albumin (BSA) monitored with fluorescence emission spectroscopy.

The evaluation of the potential biological targets of AuNRs@PEG@fenH and AuNRs@PEG@4′BrfenH focused on: (i) their interaction with calf-thymus (CT) DNA studied with UV-vis spectroscopy and DNA-viscosity measurements and via their ability to displace the reference intercalation compound ethidium bromide (EB) from its DNA-adduct explored with fluorescence emission spectroscopy, and (ii) their affinity for human serum albumin (HSA) and bovine serum albumin (BSA) monitored with fluorescence emission spectroscopy.

To evaluate the cytotoxicity of AuNRs@PEG, AuNRs@PEG@fenH and AuNRs@PEG@4′BrfenH, two breast cancer cell lines were used: the more aggressive MDA-MB-231 and the less metastatic MCF-7 cells, and two different models, two-dimensional cell cultures and spheroids. Two-dimensional cell cultures have long been a fundamental platform in cancer research, valued for their simplicity, low cost, and reproducibility, which make them essential for drug evaluation studies. However, because cells under these conditions grow as monolayers on polystyrene surfaces, they cannot fully replicate the complex architecture and microenvironmental interactions in vivo [37,38,39]. To this end, 3D cell culture systems have emerged as key models bridging the gap between conventional 2D cultures and in vivo conditions. Spheroids replicate essential features of solid tumors, including cell–cell and cell–matrix interactions, providing gradients of nutrients, oxygen, and metabolites, allowing them to more accurately mimic the microenvironment [40,41,42]. They therefore serve as a powerful tool for investigating cancer progression, treatment response, and mechanisms of drug resistance [43,44].

To our knowledge, fenamate-class NSAIDs have not previously been delivered from a PEGylated AuNR platform, and the parallel study of fenH and its 4′-bromo derivative on the same carrier isolates the effect of a single halogen substitution on both biomacromolecule affinity and cellular activity. The present work also extends our earlier naproxen-loaded AuNR system [25], which was confined to DNA-binding studies, to a combined DNA- and serum-albumin binding thermodynamic analysis coupled with evaluation in both 2D cultures and 3D spheroids—a combination that remains uncommon among AuNR-based delivery systems.

## 2. Materials and Methods

### 2.1. Materials–Instrumentation–Physical Measurements

All the chemicals used and all solvents were of reagent grade and were used as purchased from commercial sources: CT DNA, EB, BSA, HSA, NaCl, trisodium citrate, NaBH_4_ (99%), and poly(ethylene glycol) methyl ether thiol (mPEG-SH, M_n_ = 6000) from Sigma-Aldrich Co. (St. Louis, MO, USA); ascorbic acid (99.5%), cetyltrimethylammonium bromide (CTAB, 99%), sodium oleate (NaOL, 99%), HAuCl_4_·xH_2_O (99.999%, where x was estimated as 3), and AgNO_3_ (99.9995%) from Alfa Aesar (Ward Hill, MA, USA) ; and all solvents from Chemlab (Zedelgem, Belgium). Fenamic acid (fenH) was synthesized using a modified literature procedure (See Section S1 in ESI) [45,46], while the synthesis and the characterization of 4′-bromo-fenamic acid (4′BrfenH) were recently reported by our group [47].

The DNA stock solution was prepared by dilution of CT DNA into a buffer solution of pH 7.0 (15 mM trisodium citrate and 150 mM NaCl at pH 7.0) followed by stirring at 4 °C, and it was kept at 4 °C for no longer than two weeks. The stock solution of CT DNA gave a ratio of UV absorbance at 260 and 280 nm (A260/A280) in the range of ~1.90, indicating that the DNA was sufficiently free of protein contamination, and its concentration was determined by the UV absorbance at 260 nm after 1:20 dilution using ε = 6600 M^−1^ cm^−1^ [48].

TEM study was performed utilizing a FEI CM20 TEM operating (FEI Company, Hillsboro, OR, USA) at 200 kV. TEM specimens were prepared by drop casting a 3 μL droplet of AuNR nanoparticle suspension on a carbon-coated Cu TEM grid. The size of the particles was determined by “manual counting” using ImageJ software (National Institutes of Health, Bethesda, MD, USA, <https://imagej.net>).

The UV-vis spectra were recorded in the range 200–800 nm on a Jasco V-750 spectrophotometer (JASCO Corporation, Tokyo, Japan) equipped with an internal thermostat and in the range of 200–1200 nm on an Analytik jena Specord Plus spectrophotometer (Analytik Jena GmbH, Jena, Germany). Fluorescence spectra of the compounds were recorded in solution on a Hitachi F-7000 Fluorescence spectrophotometer (Hitachi, Tokyo, Japan) or on a Shimadzu RF 5301PC emission spectrophotometer (Shimadzu Corporation, Kyoto, Japan). Viscosity experiments were carried out using an ALPHA L Fungilab rotational viscometer (Fungilab S.A., Barcelona, Spain) equipped with an 18 mL LCP spindle at 100 rpm.

### 2.2. Synthesis of AuNRs

The seed solution employed for the growth of gold nanorods (AuNRs) was prepared (under aerobic conditions at RT) as follows. Initially, 5 mL of HAuCl_4_ solution (0.5 mM) was combined with 5 mL of CTAB (0.2 M). Subsequently, 1 mL of an aqueous NaBH_4_ solution (0.01 M) was rapidly injected into the mixture under vigorous stirring (750 rpm). The solution color changed from yellow to brownish-yellow, and stirring was stopped after 2 min. The resulting seed solution was allowed to age at room temperature for 30 min prior to use. For the preparation of the growth solution, 7.0 g of CTAB and 1.234 g of NaOL were dissolved in 250 mL of warm water (~50 °C) in a 1 L flask. After cooling to room temperature, 24 mL of AgNO_3_ aqueous solution (4 mM) was added, and the mixture was left undisturbed for 15 min. Then, 250 mL of HAuCl_4_ solution (1 mM) was introduced, and the solution was stirred for approximately 1.5 h until it became colorless. The pH of the growth solution at this stage was 5.8, and it was subsequently adjusted to 1.3 by the addition of 3 mL of concentrated HCl (12.1 M); acidification is essential in this binary-surfactant system, since NaOL is a basic salt and uniform nanorods are obtained only from acidic growth media (pH < 1.7). After 15 min, 1.25 mL of ascorbic acid (0.064 M) was added under vigorous stirring. Finally, 400 μL of the seed solution was introduced into the growth mixture, which was then left undisturbed overnight at 30 °C to allow the formation of AuNRs.

**AuNRs@PEG**. To remove excess CTAB, the AuNRs were purified by centrifugation at 5900 rpm (3015 RCF) three times. After each centrifugation step, the supernatant was discarded, and the pellet was redispersed in 10 mL of deionized water. For surface functionalization, 30 mg of mPEG-SH was added to 10 mL of the purified AuNR solution (O.D. = 2.9, [Au] = 260 μg/mL). The mixture was maintained at 29 °C—the set-point of the thermostatted bath, chosen so as to remain above the temperature at which CTAB crystallizes from the concentrated medium while avoiding unnecessary heating during the 24 h ligand exchange—and stirred for 24 h; the 1 °C difference with respect to the growth temperature is not mechanistically significant. The final product was collected by centrifugation at 5900 rpm for 20 min, followed by removal of the supernatant containing unreacted mPEG-SH, and finally redispersed in 10 mL of deionized water.

**Loading of fenH/4′BrfenH**. To encapsulate the fenH/4′BrfenH into the AuNRs@PEG system, 4 mg of fenH/4′BrfenH was first dissolved in 2 mL of ethanol to obtain a clear dark-brown to black solution. This solution was then added to 5 mL of the previously prepared AuNRs@PEG dispersion (O.D. = 2.4, [Au] = 239 μg/mL). The resulting mixture was sonicated in an ultrasonic water bath for approximately 20 min, leading to the formation of a pale gray emulsion. Subsequently, the final product was collected by 2 rounds of centrifugation at 5900 rpm for 20 min, followed by removal of the supernatant and finally redispersed in 5 mL of deionized water. In total, the material underwent six centrifugation/redispersion cycles (three after synthesis, one after PEGylation and two after drug loading), each comprising centrifugation at 5900 rpm (3015 RCF), removal of the supernatant and redispersion in deionized water. Residual CTAB was not quantified in this work.

### 2.3. Drug-Release Study

The release of fenH and 4′BrfenH from the nanoplatforms was monitored by fluorescence spectroscopy. Briefly, 5 mg of AuNRs@PEG@fenH or AuNRs@PEG@4′BrfenH were dispersed in 10 mL buffered solutions at pH 4.1, 6.8 and 10.1 and stirred at room temperature; at predetermined intervals up to 120 min the emission intensity of the released drug (λ_em_ = 460 nm for fenH; 415 nm for 4′BrfenH) was recorded and the cumulative release (%) was obtained from Equation (S1) using calibration curves of the corresponding free drug constructed in the same buffer. Experiments were performed in triplicate, and mean values are presented. The relative standard deviation (RSD) was estimated to ±10% for all measurements. Calibration curves were constructed independently for each buffer, and no statistically significant differences were observed between the curves. The calibration curve presented corresponds to the buffer solution at 10.1. The three buffered media were: pH 4.1, potassium hydrogen phthalate/phthalate (0.1 M); pH 6.8, NaH_2_PO_4_/Na_2_HPO_4_ (0.1 M); pH 10.1, NaHCO_3_/Na_2_CO_3_ (0.1 M). The three media are of comparable ionic strength, in the range of approximately 0.10–0.15 M. Since fenH and 4′BrfenH are practically insoluble in water, the calibration standards were prepared from a stock solution of the drug in absolute ethanol (10 mg/mL): aliquots of 25, 50, 250 and 500 µL were transferred to separate 50 mL volumetric flasks, 475, 450, 250 and 0 µL of absolute ethanol were added, respectively, and the flasks were made up to volume with the corresponding buffered medium, affording standards of 0.005, 0.01, 0.05 and 0.1 mg/mL at a constant ethanol content of 1.0% (*v*/*v*). The release samples themselves contained no added ethanol. The full protocol and equations are given in Section S2 of the ESI.

### 2.4. Study of the Interaction of the AuNRs with Biomacromolecules

The AuNRs were initially dissolved in a buffer solution of pH = 7 (150 mM NaCl and 15 mM trisodium citrate) at a concentration of 0.01 mg/mL. CT DNA and the albumins were also dissolved in same buffer solution (150 mM NaCl and 15 mM trisodium citrate, pH = 7).

The interaction of the AuNRs with CT DNA was examined thoroughly with UV-vis spectroscopy for three different temperatures (291 K, 300 K, and 310 K) and viscosity measurements, and via competitive studies with EB monitored by fluorescence emission spectroscopy. The affinity of the AuNRs for albumins (BSA or HSA) was evaluated through tryptophan fluorescence quenching experiments. All the specific protocols and relevant equations involved in the in vitro study of the interaction of the AuNRs with biomacromolecules are summarized in the ESI (Sections S2 and S3).

### 2.5. Cell Cultures

The MDA-MB-231 and MCF-7 breast cancer cell lines were obtained from the American Type Culture Collection (ATCC). The cells were cultured at 37 °C in a humidified atmosphere of 5% CO_2_. The cell culture medium was Dulbecco’s Modified Eagle Medium (DMEM, LM-D1110/500, Biosera, Nuaillé, France) and supplemented with 10% fetal bovine serum (FBS, FB-1000/500, Biosera, Nuaillé, France), antimicrobial agents (100 IU/mL penicillin, 100 µg/mL streptomycin, 10 µg/mL gentamycin sulfate and 2.5 µg/mL amphotericin B), and 2 mM L-glutamine (Biosera, Nuaillé, France). The cells were harvested using trypsin–EDTA in PBS (LM-T1706/500, Biosera, Nuaillé, France). All experiments were conducted in serum-free conditions using three biological replicates.

### 2.6. Cytotoxicity Assays

#### 2.6.1. Two-Dimensional Cell Cultures

MDA-MB-231 and MCF-7 were seeded in 24-well plates at a density of 30.000–40.000 and cultured to 70–80% confluency. The synthesized compounds were dissolved in sterile ddH_2_O, and appropriate serial dilutions were prepared for further use. Cells were cultured overnight in serum-free medium (0% FBS) to establish controlled experimental conditions, allowing the effects of the drug to be assessed without interference from serum-derived components. Then cells were incubated with AuNRs@PEG, AuNRs@PEG@fenH and AuNRs@PEG@4′BrfenH at different concentrations (1, 50, and 200 μg/mL) for 24 h, as reported in previous studies [49]. The adherent cells were subsequently harvested and counted manually through a Neubauer hemacytometer, and the cytotoxicity was estimated as a percentage of living cells relative to non-treated control cells ± standard deviation (SD) of experiments in triplicate. The graphs were created with GraphPad Prism 8 (GraphPad Software, San Diego, CA, USA).

#### 2.6.2. Three-Dimensional Spheroids

Both cell lines were seeded in 96-well round-bottom plates with ultra-low adhesive properties (911606, SPL Life Sciences, Pocheon, Republic of Korea) at a density of 10.000 and incubated with complete cell culture medium (10% FBS) for 3 days till the spheroids were formed [25,42,49]. Following an overnight starvation in serum-free medium (0% FBS), the cells were incubated with AuNRs@PEG, AuNRs@PEG@fenH and AuNRs@PEG@4′BrfenH at different concentrations (1, 50, and 200 μg/mL) for 24 h. The spheroids were monitored after 24 h using a contrast microscope (OLYMPUS CKX41, Center Valley, PA, USA) connected to a digital camera (QImaging MicroPublisher 3.3RTV, Preston, Australia). The obtained photos were further analyzed using ImageJ (1.50b Launcher Symmetry Software, LOCI, University of Wisconsin, Madison, WI, USA) to quantify the spheroid perimeter after 24 h of treatment in comparison with the control samples (untreated cells). The latter area was measured in inches [2] and subsequently normalized to the control values, expressed as percentages. As a result of this normalization, the control was set to 100%, and the units were effectively canceled. The 3D tumor models were utilized to highlight their relevance as tools for drug evaluation, given their ability to closely resemble the complexity of in vivo tumors and their microenvironment.

## 3. Results and Discussion

### 3.1. Synthesis and Characterization

**Figure 2:**
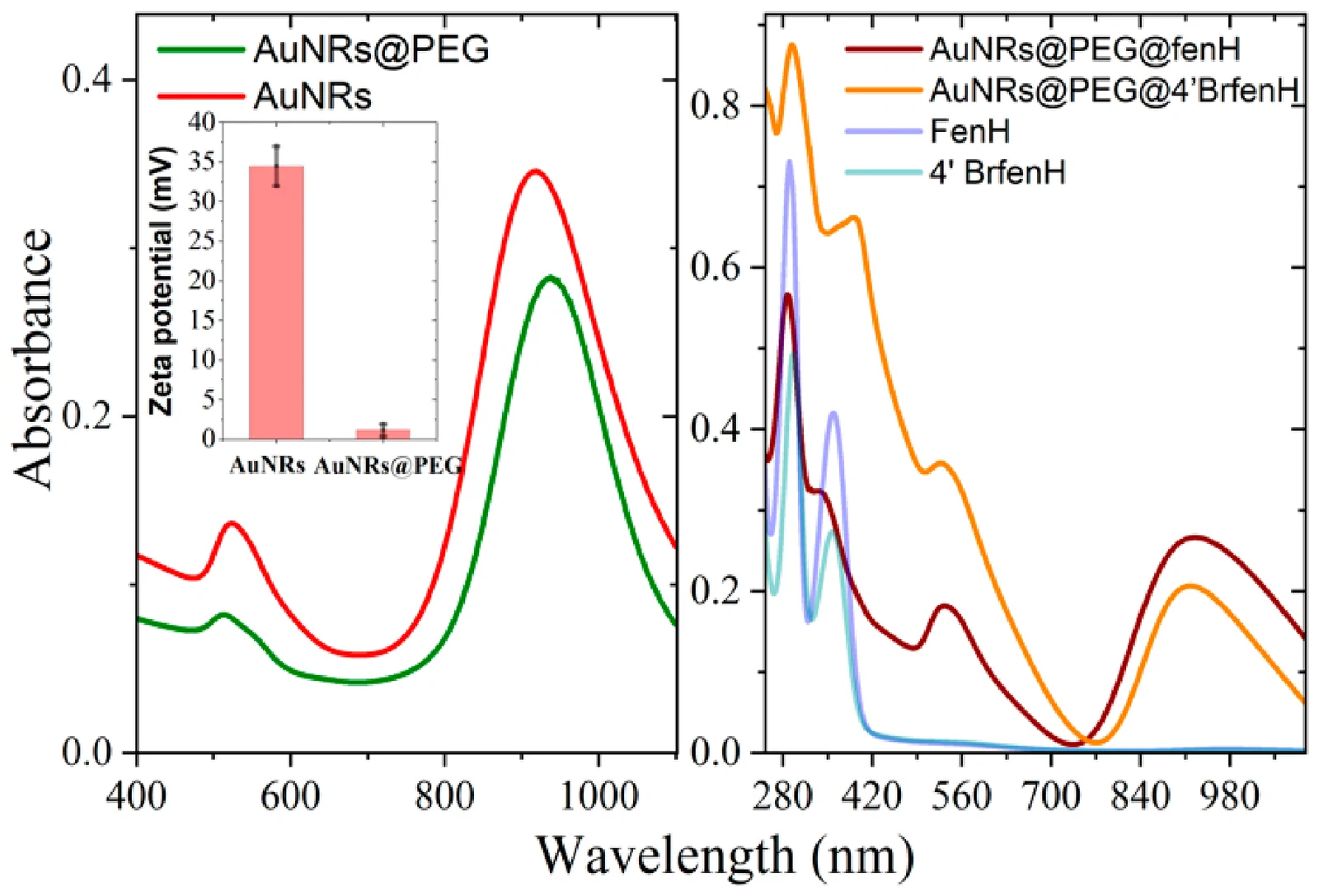
(**Left**) UV-vis-NIR absorption spectra of aqua solutions of AuNRs and AuNRs@PEG. The inset shows the ζ-potential measurements of AuNRs and AuNRs@PEG. (**Right**) UV-vis-NIR absorption spectra of aqua solutions of AuNRs@PEG@fenH and AuNRs@PEG@4′BrfenH along with the spectra of the NSAIDs fenH and 4′BrfenH.

AuNRs were synthesized using a seed-mediated growth method with binary surfactant mixtures of CTAB/NaOL, following a modified protocol [50]. The absorption spectrum exhibited two distinct peaks at approximately 518 nm (transverse SPR, TSPR) and 920 nm (longitudinal SPR, LSPR) and is shown in Figure 2. The position of the LSPR peak correlates with the aspect ratio of the AuNRs, as described by Equation (1) [51], yielding a calculated aspect ratio (AR) of 5.3.

λ_max_(nm) = 95R + 420 (1)

Evidence for effective PEGylation of the AuNRs includes a 12 nm red shift in the LSPR band of the absorption spectrum for AuNRs@PEG dispersions, as well as a reduction in ζ-potential from 34.8 mV (CTAB-covered AuNRs) to approximately 1.2 mV for AuNRs@PEG (Figure 2, inset) [52]. The pH-dependence of the ζ-potential in the release buffers, and the hydrodynamic diameter and polydispersity index by DLS, were not determined in this study; these characterizations are required in order to describe the colloidal behavior of the platform in the release media and are identified as future work. It may nevertheless be noted that for the closely related system of pegylated ferrite nanoparticles loaded with mefenamic acid, a point of zero charge at pH ≈ 2.5 has been reported (see Section 3.2). As this value lies well below the pH of all three-release media, a fenamate-loaded pegylated particle would be expected to bear a net negative charge at pH 4.1, 6.8 and 10.1, becoming progressively more negative as the carboxylic group is deprotonated. This expectation is drawn from a literature analog with a different core and a different PEG chemistry and is offered only as a qualitative guide; it does not replace measurements on the present materials. The loading of AuNRs with the NSAIDs can be verified by the UV-vis-IR spectra of the final hybrids, AuNRs@PEG@fenH and AUNRs@PEG@4′BrfenH, where additional charge transfer (CT) bands are present related to the fenH and 4′BrfenH acids (Figure 2, right). In the same figure, the UV-Vis spectra of the relevant acids are also presented. The effective loading of the AuNRs@PEG is further confirmed by the 4 and 8 nm red shifts in the LSPR peaks of AuNRs@PEG@fenH and AuNRs@PEG@4′BrfenH, respectively.

**Figure 3:**
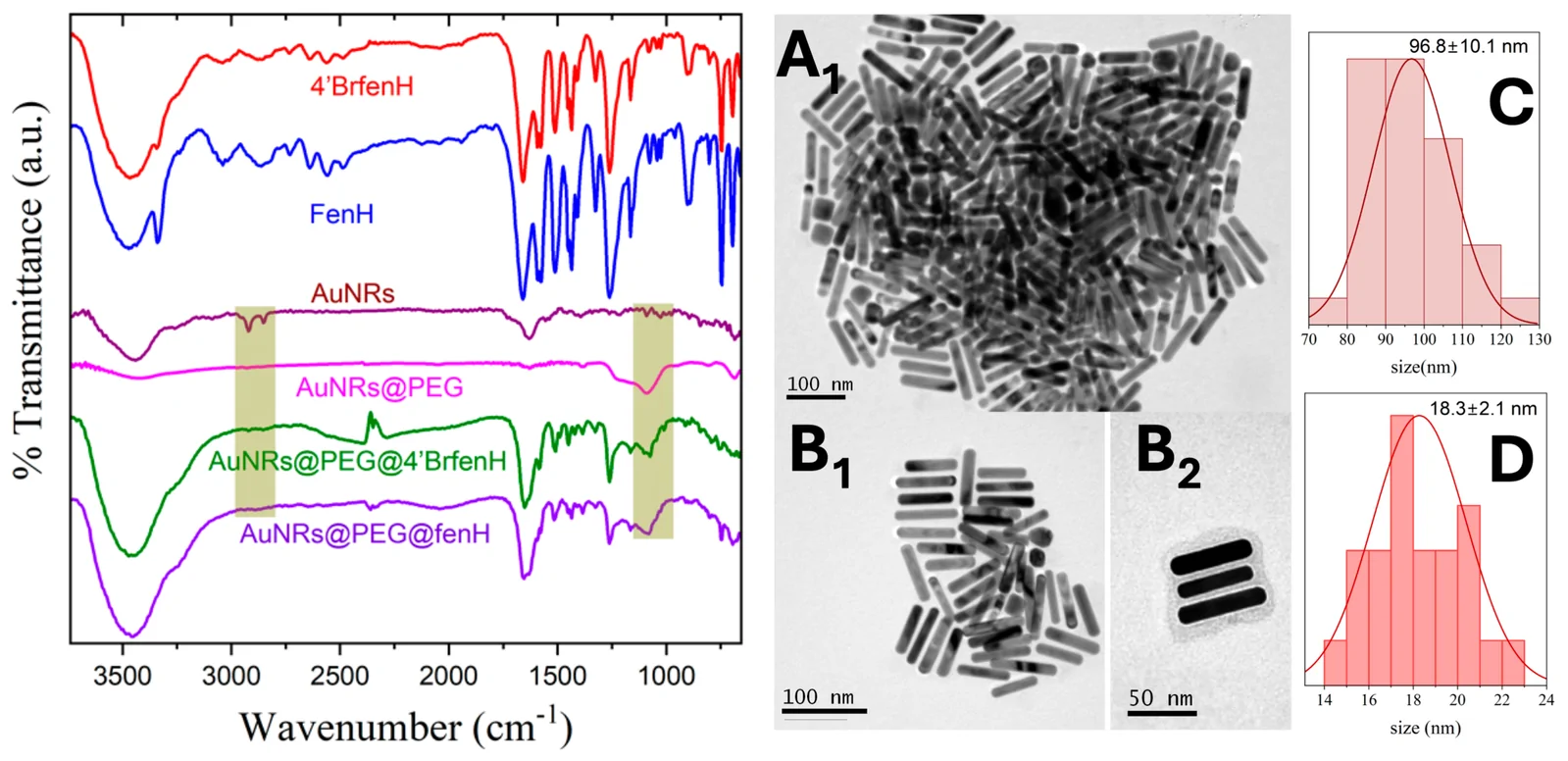
(**Left**) FT−IR spectra of the NSAIDs fenH (blue line) and 4′BrfenH (red line), as well as the AuNRs (wine line), AuNRs@PEG (pink line), AuNRs@PEG@fenH (violet line) and AuNRs@PEG@4′BrfenH (green line). The highlighted regions are related to the effective PEGylation of the AuNRs (see text for details). (**Right**) (**A1**) TEM images of the AuNRs; (**B1**,**B2**) TEM images of the AuNRs@PEG, where (**B2**) shows the thin coverage of PEG. (**C**,**D**) Gaussian distribution of sizes concerning the length and width of the AuNRs.

Transmission electron microscopy (TEM) images showed rod-shaped gold nanoparticles with a length of 96.8 ± 10.1 nm, a width of 18.3 ± 2.1 nm, and an aspect ratio (AR) of approximately 5.4, which is consistent with the value obtained from UV-vis-NIR measurements (Figure 3A1,C,D). In the same figure, the pegylated AuNRs are shown (Figure 3B1,B2), where a faint low-contrast halo of approximately 3 nm is discernible at the particle edge (arrows). Because PEG is essentially electron-transparent, this thickness is an estimate derived from the edge contrast rather than a direct measurement, and unambiguous visualization would require negative staining or cryo-TEM. The presence of the PEG shell is independently supported by the 12 nm LSPR red shift, by the change in ζ-potential from +34.8 to ≈+1.2 mV, and by the C–O–C ether band at 1108 cm^−1^.

Further confirmation of effective PEGylation of the AuNR surface was obtained using FT-IR spectroscopy. The IR spectra of AuNRs@PEG@fenH and AuNRs@PEG@4′BrfenH (Figure 3) display the multiple C-O-C ether vibration modes at 1108 cm^−1^ of mPEG-SH. Successful ligand exchange between CTAB and mPEG-SH during PEGylation is indicated by the disappearance of the characteristic bands at 2918 and 2848 cm^−1^, corresponding to the asymmetric CH2/CH3 groups of CTAB [53,54]. Additionally, absorption in the ~1640–1724 cm^−1^ region, which is absent from the drug-free AuNRs@PEG control, is consistent with the presence of the carboxylic acid group of the loaded drug. This spectral window is congested, since carbonyl and carboxylate stretching modes overlap with aromatic C=C and amine deformation modes, and this feature is therefore not conclusive on its own; the evidence for successful loading rests on its convergence with the new charge-transfer bands in the UV-vis spectra, the 4 and 8 nm LSPR red shifts, and the quenched drug fluorescence that is recovered upon release. These features are shown in Figure 3. For comparison, the FT-IR spectra of the AuNRs, AuNRs@PEG and the NSAID acids are also presented in the same Figure.

### 3.2. Drug-Release

**Figure 4:**
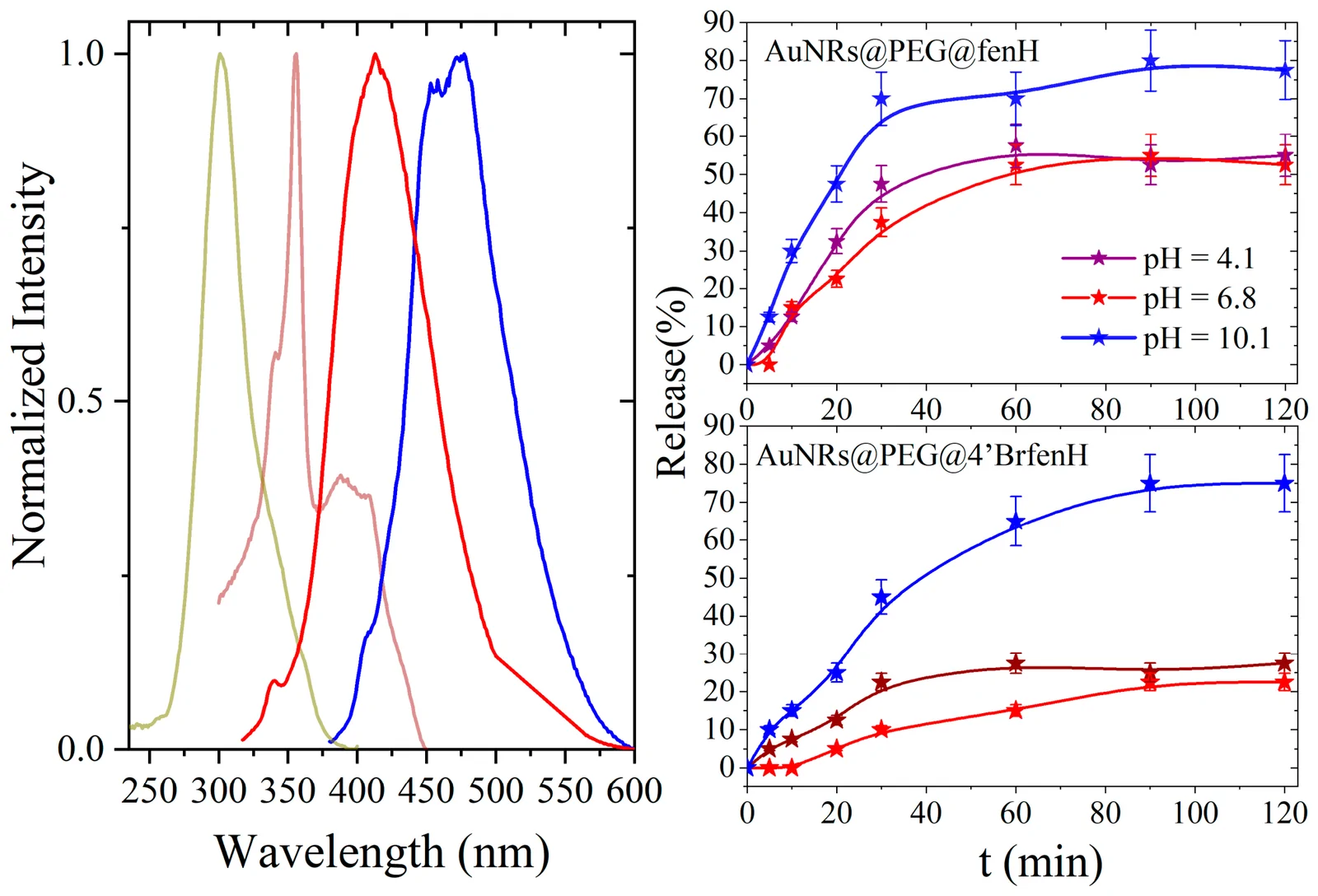
(**Left**) Fluorescence (excitation (brown line) and emission (blue line)) spectra of AuNRs@PEG@fenH (with λem = 460 nm and λexc = 380 nm, respectively) and fluorescence (excitation (green line) and emission (red line)) spectra of AuNRs@PEG@4′BrfenH (with λem = 415 nm and λexc = 301 nm, respectively). (**Right**) Drug-release behavior of AuNRs@PEG@fenH or AuNRs@PEG@4′BrfenH in three buffered solutions (pH = 4.1, 6.8 and 10.1). Release data are given as mean values of three independent experiments; error bars represent the estimated relative standard deviation of ±10% applying to all measurements.

**Scheme 1:**
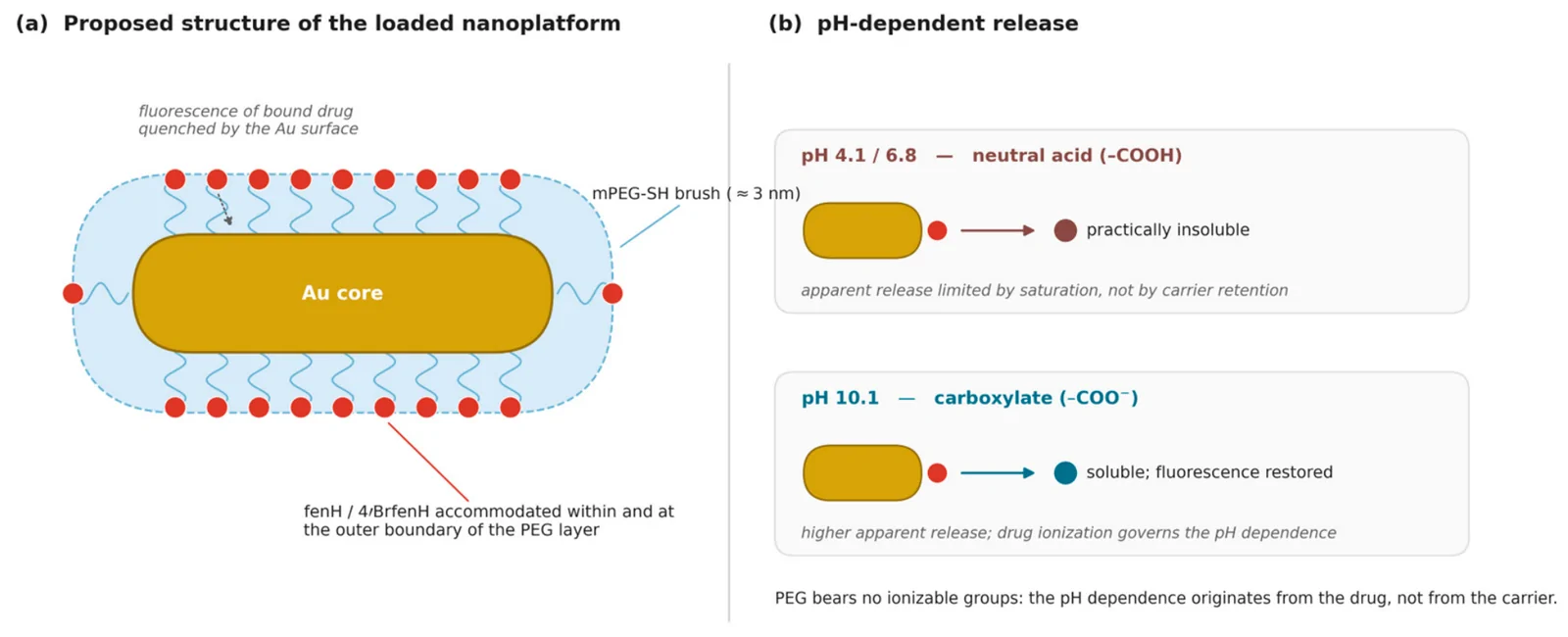
Proposed structure of the fenamate-loaded AuNRs@PEG nanoplatform and of the pH-dependent release step. (**a**) The mPEG-SH brush (≈3 nm) surrounds the Au core, with fenH or 4′BrfenH accommodated within and at the outer boundary of the polymer layer; the fluorescence of the bound drug is quenched by the gold surface and is recovered upon release. (**b**) At pH 4.1 and 6.8, the drug is released in its neutral, practically insoluble form, so that the apparent release is limited by saturation rather than by retention by the carrier, whereas at pH 10.1 the soluble carboxylate is formed and a higher apparent release is recorded. The model is consistent with the spectroscopic data and is not a directly determined structure.

The excitation and emission spectra of an aqueous suspension of AuNRs@PEG@fenH and AuNRs@PEG@4′BrfenH are presented in Figure 4. As a result of the fluorescence properties of fenH and 4′BrfenH [47], their successive loading on the pegylated surfaces of gold NRs was confirmed with fluorescence spectroscopy. FenH presented an excitation and an emission band with λmax at 355 nm and 470 nm, respectively, with a large Stokes shift of 115 nm, while in the spectra of 4′BrfenH these bands were observed at 301 nm and 413 nm, respectively, with a Stokes shift of 112 nm. From the maximum emission intensity and the calibration curves (Figure S1), the quantity of fenH and 4′BrfenH in this dispersion is calculated at 0.04 mg/mL (0.04 g/L), the dispersion containing 0.5 g/L of nanoplatform, and their loading capacity (LC) is calculated at around 80 mg/g, while the encapsulation efficiency (EE) is 5%. It should be noted that the representative excitation and emission spectra reproduced in Figure 4 (left) were recorded on a separate, more dilute aliquot at a nanoplatform concentration of 0.1 g/L, chosen so as to afford a suitable signal level; that value refers to the spectra shown and is not the concentration from which the loading was determined. The LC and EE were defined as LC (mg/g) = (mass of drug loaded/mass of nanoplatform) × 1000 and EE (%) = (mass of drug loaded/mass of drug added) × 100, and were evaluated on a single, consistent mass basis. The encapsulation efficiency of ≈5% is low, which is expected for this architecture. The nanoplatform is not an encapsulating core–shell reservoir: the mPEG-SH brush is only a few nanometres thick, so the accessible loading volume is intrinsically small, and the drug is offered in large excess (4 mg per 5 mL of dispersion) with the unbound fraction removed in two centrifugation/redispersion cycles. A low EE combined with a moderate loading capacity (≈80 mg/g) is therefore the anticipated outcome. As regards the mode of immobilization, the fluorescence of the loaded drug is strongly quenched in the intact nanoplatform and is recovered upon release, which requires the fenamate to reside within the quenching distance of the gold surface, that is, accommodated within and at the outer boundary of the PEG layer rather than free in solution. A proposed model of the loaded nanoplatform and of the release step is shown in Scheme 1; it is a model consistent with the spectroscopic data and not a directly determined structure.

In order to monitor the pH-sensitivity of targeted drug delivery, three representative pH values (4.1, 6.8 and 10.1) were chosen, and the drug release (Equation (S1)) was calculated for different times intervals up to 2 h. At acidic (4.1) or neutral pH (6.8), the release profile indicates a release up to a maximum of 55% for fenH and 27% for 4′BrfenH within the first 120 min, while a higher percentage of release (77.5% for fenH and 75% for 4′BrfenH) was observed at pH = 10.1 for the same time (Figure 4, right). The strong pH dependence of the release is attributed principally to the acid–base speciation of the fenamates themselves: both drugs are weak acids that are practically insoluble in their neutral form and become markedly more soluble upon deprotonation, so that the higher apparent release at pH 10.1 largely reflects the solubility of the released drug rather than an intrinsic pH response of the PEGylated carrier, which bears no ionizable groups. Matched dissolution experiments on the free drugs in the same three buffers were not performed, and in their absence the relative contributions of pH-dependent drug solubility and of drug–carrier association cannot be deconvoluted from the present data. The release curves should therefore be read as indicative, comparative trends between the two drugs recorded under identical non-sink conditions at room temperature over 120 min, and not as quantitative release kinetics. This behavior is opposite to acid-burst carbonate carriers and is consistent with a platform suited to low-pH storage and neutral/alkaline release.

It is instructive to place the measured release levels on an absolute concentration scale. Dispersing 5 mg of nanoplatform in 10 mL corresponds, at a loading capacity of ≈80 mg/g, to a maximum attainable concentration of ≈40 µg/mL upon complete release. The plateaus recorded at pH 4.1 and 6.8 therefore correspond to approximately 22 µg/mL for fenH and 11 µg/mL for 4′BrfenH, as against approximately 31 and 30 µg/mL at pH 10.1. Fenamates in their neutral form are practically water-insoluble, saturation solubilities of only a few tens of µg/mL at most being reported in plain aqueous media for the closely related mefenamic acid, with a further decrease below the pKa (≈4.3) and a steep increase upon deprotonation [55,56]. The concentrations attained at pH 4.1 and 6.8 thus lie at or above the solubility expected for the neutral acids, whereas those measured at pH 10.1 are readily accommodated by the much more soluble carboxylate. The plateaus observed under acidic and neutral conditions are therefore most plausibly saturation-limited rather than indicative of retention by the carrier. It should further be noted that the buffer species and the ionic strength, and not pH alone, can modify the hydration and conformation of oligo(ethylene glycol) layers such as the mPEG-SH shell employed here [57]; since the three media differ in composition as well as in pH, a buffer-specific contribution to the observed profiles cannot be excluded. These values follow directly from the nanoplatform concentration of 0.5 g/L employed in the release experiments (5 mg in 10 mL), which is the same as that of the dispersion from which the loading capacity was determined.

Two features of the analytical procedure should be noted in this connection. The calibration standards were obtained by dilution of an ethanolic stock into the buffered media, a solvent-shift procedure which is known to afford transiently supersaturated solutions of poorly soluble compounds; the linearity of the calibration lines up to 0.1 mg/mL therefore reflects the concentration of drug present in solution during the measurement and does not establish the equilibrium solubility of the free acids in these media. Drug dissolving from a solid-supported state, by contrast, cannot exceed the equilibrium solubility, so that the two situations are not equivalent. In addition, the standards contained 1.0% (*v*/*v*) ethanol, which the release samples did not; although this proportion is small, a minor difference in the fluorescence response between standards and samples cannot be excluded. These considerations are consistent with the interpretation given above, but they do not replace the matched dissolution controls, which remain necessary.

It has been shown elsewhere [54,55] that, for the case of the related mefenamic acid, the maximum of the dissolution rate is close to pH = 9.0, while for the case of pegylated ferrite NPs loaded with mefenamic acid, the point of zero charge (PZC) was recorded at pH ≈ 2.5 and negative zeta potential values were recorded in the range of −18 to −22 mV. This observation is informative beyond the dissolution behavior. Poly(ethylene glycol) carries no ionizable groups and cannot by itself generate a point of zero charge as low as pH 2.5, whereas the bare ferrite surface is governed by hydroxyl groups and lies at appreciably higher pH. The displacement of the point of zero charge to strongly acidic values in the drug-loaded material is therefore attributable to the carboxylic groups of the fenamate. This indicates, on the one hand, that the drug is located at or near the electrokinetic surface, in agreement with the model proposed in Scheme 1, and, on the other, that in nanoplatforms of this class the pH-active component is the drug rather than the polymer coating, as adopted in the interpretation given above. Similar pH-sensitivity of the drug release was also observed for an AuNR nano-platform loaded with naproxen [26] and PEGylated polypeptide vesicles/polymeric capsules [58,59], indicating suitability for drug delivery systems that are able to store the drugs under a low-pH environment, e.g., the stomach, and release efficiently under neutral and alkaline environments, e.g., intestines.

The relevance of this profile to a tumor setting requires comment. The extracellular microenvironment of solid tumors is mildly acidic (pH ≈ 6.5–7.0) rather than alkaline, and the present system is therefore not one whose release is triggered by the tumor microenvironment; no such claim is made here. Appreciable release is nevertheless already recorded at pH 6.8, which approximates the extracellular tumor pH, amounting to 55% for fenH and 27% for 4′BrfenH within 120 min. Furthermore, since these experiments were performed under non-sink conditions, in which the released drug accumulates until saturation is approached, the values measured at pH 4.1 and 6.8 are likely to underestimate the release attainable under physiological sink conditions, where continuous removal of the drug by protein binding—the affinity of the present nanoplatforms for serum albumins is documented in Section 3.4—and by cellular uptake would prevent saturation from being reached. The alkaline-biased profile is accordingly best regarded as a consequence of the solubility of the drug rather than as a targeting mechanism, and it is of more direct relevance to compartments of neutral-to-alkaline pH, such as the intestinal tract, than to the acidic tumor microenvironment. Verification of these expectations requires release measurements under sink conditions and in biologically relevant media, which were not undertaken here.

Such pronounced release observed in the first 1 h followed by slow release is consistent with an initial surface-desorption step followed by diffusion-controlled release; formal kinetic modeling (e.g., Korsmeyer–Peppas) would be required to confirm the mechanism and is identified as future work [58,59,60,61].

### 3.3. Interaction of AuNRs with CT DNA

**Figure 5:**
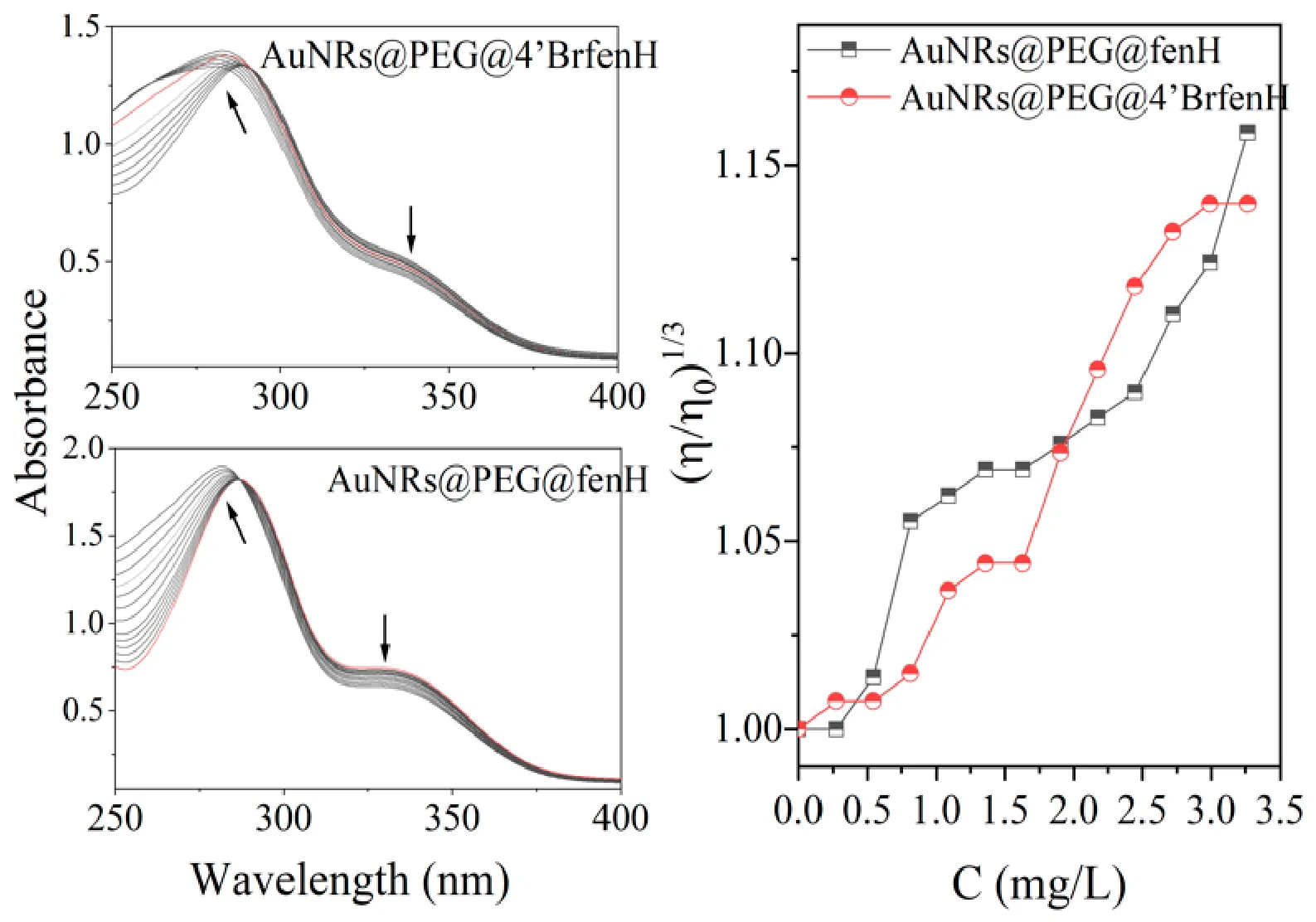
(**Right**) UV-vis spectra of AuNRs@PEG@fenH (0.01 mg/mL) and AuNRs@PEG@4′BrfenH (0.01 mg/mL) in buffer solution (150 mM NaCl and 15 mM trisodium citrate at pH = 7.0) in the presence of increasing amounts of CT DNA. The spectra were recorded at 22 °C. The arrows show the changes upon the addition of CT DNA solution. (**Left**) Relative viscosity (η/η_0_)^1/3^ of CT DNA (0.1 mM) in buffer solution (150 mM NaCl and 15 mM trisodium citrate at pH 7.0) in the presence of AuNRs at increasing concentrations.

DNA is a usual target for diverse drugs including those bearing antibacterial, antiviral, and anticancer efficacy [62]. Within this context, the interaction of potentially bioactive compounds with DNA is often monitored complementarily to cytotoxic studies or other possible mechanisms or applications. The interaction of AuNRs@PEG@fenH and AuNRs@PEG@4′BrfenH with CT DNA was investigated directly by UV-vis spectroscopy and viscosity measurements and by evaluating their ability to displace EB from the EB–DNA adduct. UV-vis spectroscopy was initially employed to gain information on the mechanism and the intensity of this interaction between CT DNA and the AuNRs (AuNRs@PEG@fenH and AuNRs@PEG@4′BrfenH). For this purpose, the UV-vis spectra of the AuNRs were recorded in the presence of increasing amounts of CT DNA (Figure 5). In both cases, the two UV bands observed in the regions 287–289 nm and 328–335 nm showed slight hypochromism and hyperchromism, respectively (Table 1). Similar behavior with much more intense changes was observed in the spectra of the free fenH and 4′BrfenH (Table 1) [47]. Such features may indicate the interaction of AuNRs with CT DNA, but the possible interaction mode may not be concluded safely.

The DNA-binding constants of AuNRs (K_b_) were calculated with the Wolfe–Shimer equation (Equation (S2)) [63] and the corresponding plots [DNA]/(ε_A_ − ε_f_) versus [DNA] (Figure S2). At room-temperature, AuNRs@PEG@fenH presents lower K_b_ values than AuNRs@PEG and free fenH. On the contrary, AuNRs@PEG@4′BrfenH presents higher K_b_ values than AuNRs@PEG and free 4′BrfenH (Table 1). In addition, AuNRs@PEG@4′BrfenH presents similar or less tight interaction with CT DNA than the recently reported Co(II) complexes with 4′Brfen^−^ ligands (Table S1) [47].

Deeper information regarding the possible forces developed upon interaction of the AuNRs with CT DNA can be obtained by investigating the changes in K_b_ values determined for three different temperatures (295 K, 302 K, and 309 K). As noted in Table 2, the increase in temperature results in higher K_b_ values for both AuNRs studied herein. Such behavior is similar with that previously reported for the analog AuNRs@PEG@NAP (where NAP is the NSAID naproxen) and different from that of the host AuNR, AuNRs@PEG [25].

The most common interaction forces developed between a bioactive compound and a biomolecule are hydrophobic forces, electrostatic interactions, van der Waals interactions, and hydrogen bonds [64,65]. According to the literature, these interactions may affect the enthalpy change (ΔH) and the entropy change (ΔS) of the interactions. More specifically, hydrophobic forces result in positive values of ΔH and ΔS (ΔH > 0 and ΔS > 0); the presence of van der Waals interactions and hydrogen bonds leads to negative ΔH and ΔS values (ΔH < 0 and ΔS < 0), while the combination of ΔH < 0 and ΔS > 0 is found when electrostatic interactions are developed [66,67]. The K_b_ values of Table 2 obtained for three different temperatures were used to calculate the ΔH and ΔS values with the van’t Hoff equation (Equation (S3)) and the plots of ln(K_b_) versus (1/T) (Figure S3), where –ΔH/R is the slope of the fitting line, and ΔS/R is the intercept (R is the universal gas constant), while the ΔG values were obtained from the Gibbs–Helmholtz equation (Equation (S4)).

For both AuNRs, both ΔH and ΔS bear positive values, suggesting the existence of hydrophobic forces between AuNRs and CT DNA, stabilized by π–π stacking interactions, which may be explained by an intercalative interaction [66,67], as reported in the case of AuNRs@PEG@NAP. The negative ΔG values for both AuNRs show a spontaneous interaction with CT DNA [65,68,69].

Studying DNA viscosity is a complementary technique to clarify/verify the DNA-interaction mode of a compound, due to the sensitivity of the DNA viscosity to relative DNA-length changes [70]. Within this context, the viscosity of CT DNA solution (0.1 mM) was measured upon addition of increasing amounts of AuNRs, and an increase in the relative DNA viscosity was observed (Figure 5). Such an increase in DNA viscosity may be attributed to an increase in the relative DNA length, probably resulting from an increase in the separation distance of DNA base pairs when hosting an intercalating compound [70].

**Figure 6:**
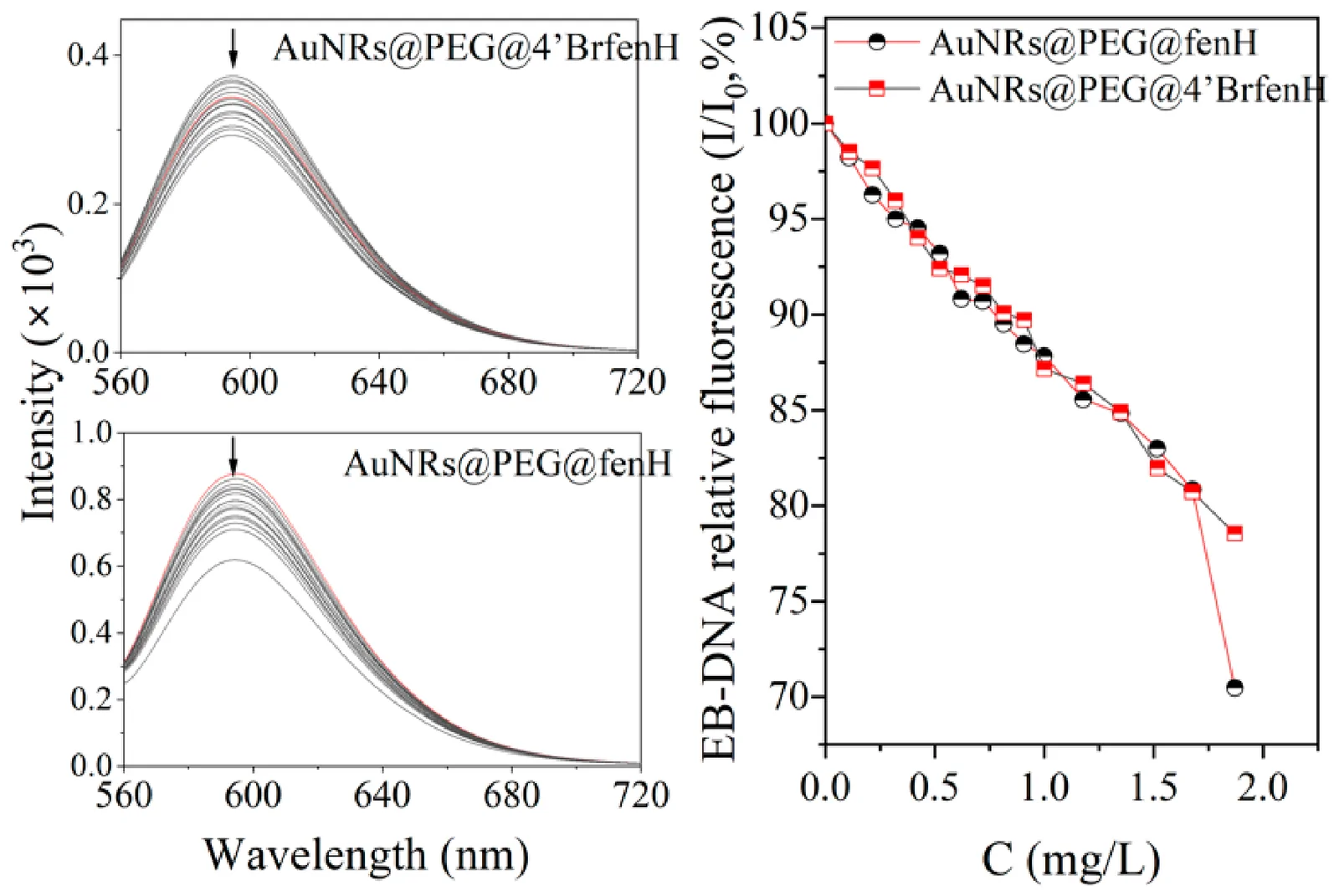
(**Left**) Fluorescence emission spectra (λ_exc_ = 540 nm) for EB–DNA adduct ([EB] = 20 μM, [DNA] = 26 μM) in buffer solution (150 mM NaCl and 15 mM trisodium citrate at pH = 7.0) in the absence and presence of increasing amounts of AuNRs@PEG@fenH and AuNRs@PEG@4′BrfenH. The arrow shows the changes in intensity upon increasing amounts of AuNRs@PEG@fenH and AuNRs@PEG@4′BrfenH. (**Right**) Plot of EB–DNA relative fluorescence intensity at λ_emm,max_ = 593 nm (I/I_0_, %) versus the concentration of AuNRs@PEG@fenH and AuNRs@PEG@4′BrfenH (up to 70.5% of the initial EB–DNA fluorescence intensity for AuNRs@PEG@fenH (black line), and 78.5% for AuNRs@PEG@4′BrfenH (red line)).

The reported DNA-binding constant (K_b_ = 1.23 (±0.07) × 10^5^ M^−1^) [71] of the typical DNA-intercalator ethidium bromide (EB) is close to the values reported for the AuNRs studied herein. Since a DNA-intercalator may displace EB from the EB–DNA adduct, the EB-displacing ability of the AuNRs was studied with fluorescence emission spectroscopy. The quenching of the intense fluorescence emission band observed for the EB-DNA adduct at 593 nm, upon excitation of its solution at 540 nm, may reveal this displacing ability [72]. For this purpose, the changes in the EB-DNA emission band of a solution containing 20 µM EB and 26 µM CT DNA pretreated for 1 h were monitored upon addition of solutions of AuNRs (Figure 6). The quenching resulting from the displacement of EB from the EB–DNA was moderate and less intense than that observed for AuNRs@PEG [26] or free fenH and 4Br’fenH [47] (Table 3).

The K_SV_ constants (Table 3) of the AuNRs were calculated with the Stern–Volmer Equation (S5) and the corresponding Stern–Volmer plots (Figure S4) and may reveal the EB-displacing inability of the AuNRs. The corresponding EB–DNA quenching constants (Kq) were derived with Equation (S6) [72] when applying the value of 23 ns as the fluorescence lifetime (τ_0_) of the EB–DNA system [73]. The Kq values of the AuNRs (Table 3) are higher than those of free fenH and 4Br’fenH [47], as well as of AuNRs@PEG and AuNRs@PEG@NAP [25] (Table 3), and suggest the existence of a static quenching mechanism for the EB–DNA fluorescence [72], revealing the displacement of EB indirectly.

### 3.4. Interaction of the AuNRs with Albumins

**Figure 7:**
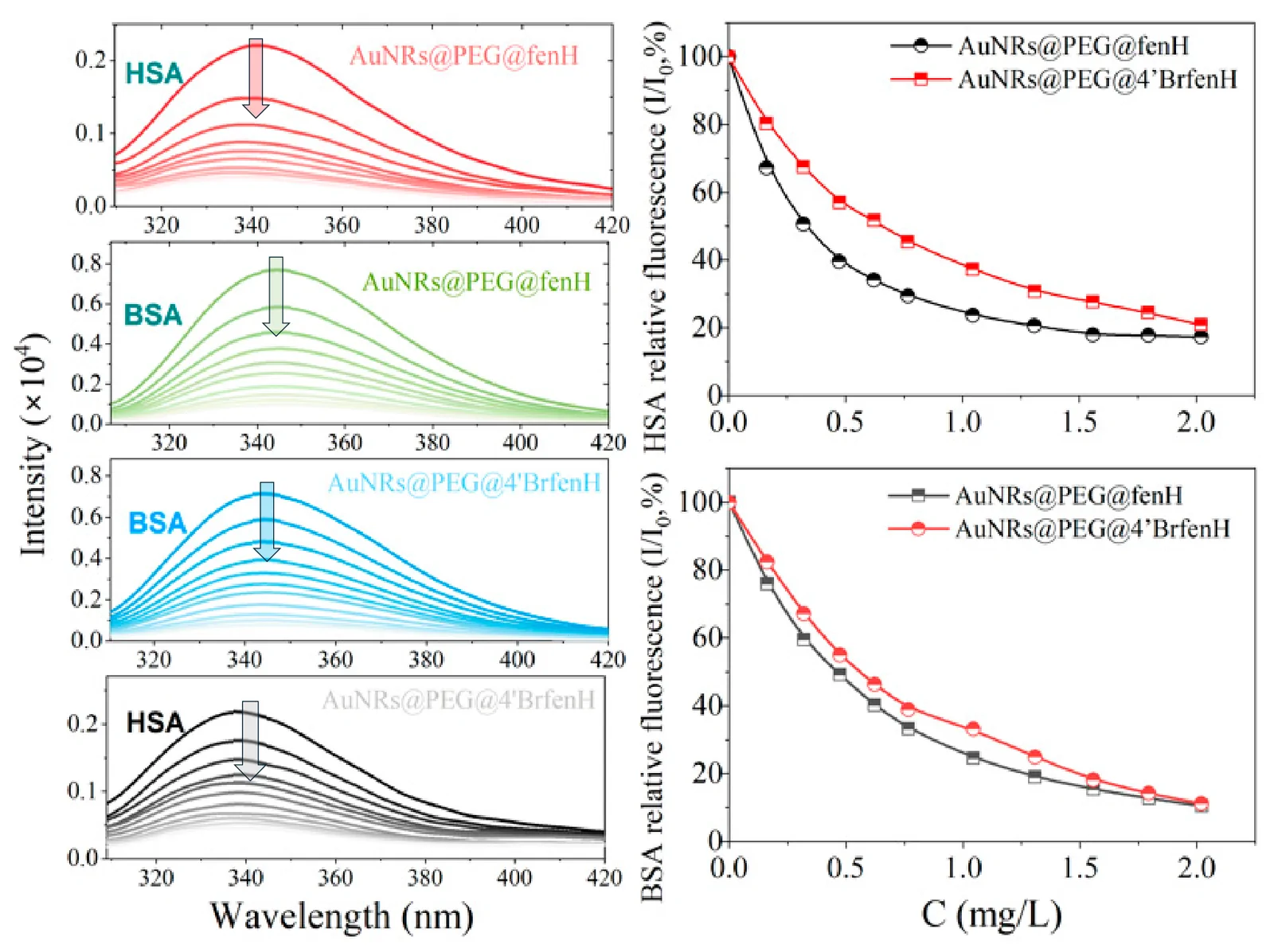
(**Left**) Fluorescence emission spectra (λ_exc_ = 295 nm) for BSA ([BSA] = 3 μM) and HSA ([HSA] = 3 μM) in buffer solution (150 mM NaCl and 15 mM trisodium citrate at pH 7.0) in the absence and presence of increasing amounts of AuNRs@PEG@fenH and AuNRs@PEG@4′BrfenH. The arrow shows the changes in intensity upon increasing amounts of AuNRs@PEG@fenH. (**Right**) Plot of relative fluorescence emission intensity of BSA at λ_em,max_ = 345 nm (I/I_0_, %) versus the concentration of AuNRs@PEG@fenH and AuNRs@PEG@4′BrfenH (up to 10.5% of the initial BSA fluorescence for AuNRs@PEG@fenH (black line), and 11.1% for AuNRs@PEG@4′BrfenH (red line)). Plot of relative fluorescence emission intensity of HSA at λ_em,max_ = 340 nm (I/I_0_, %) versus the concentration of AuNRs@PEG@fenH and AuNRs@PEG@4′BrfenH (up to 17.3% of the initial HSA fluorescence for AuNRs@PEG@fenH (black line), and 20.8% for AuNRs@PEG@4′BrfenH (red line)).

The interaction of the most important plasma protein that participates in the transportation of metal ions, drugs, and small molecules to their biological targets [72], serum albumin (SA), with potentially bioactive compounds is a necessary study to monitor their binding strength to their potential carriers or to investigate differentiation of biological properties or mechanisms of action [74]. Within this context, the interaction of the AuNRs with the homologs HSA and BSA was studied with fluorescence emission quenching experiments (Figure 7), applying excitation at λ = 295 nm [72]. The addition of the AuNRs at increasing amounts into the albumin solutions resulted in intense quenching (up to 87.5% in Figure 7 and Table 4) of the respective emission band (345 nm for BSA and 340 nm for HSA). The inner-filter effect was also checked (Equation (S7)) [75], and it was found negligible to affect the measurements.

The interaction of the AuNRs with the albumins was further evaluated by the determination of the SA-quenching constants (Kq) with the Stern–Volmer quenching equation (Equations (S5) and (S6)) [72], where the fluorescence lifetime, τ_0_, of tryptophan in SA is 10^−8^ s [76] and the corresponding Stern–Volmer plots are shown in Figures S5 and S6. The obtained Kq values (Table 4) indicate the existence of a static quenching mechanism and therefore verify the interaction of the AuNRs with the SAs. It may be noted that the Kq values of AuNRs@PEG@fenH and AuNRs@PEG@4′BrfenH are higher than those of free fenH and 4′BrfenH [47], respectively, as well as of AuNRs@PEG and AuNRs@PEG@NAP [25].

The SA-binding constants (K) of the AuNRs were calculated with the Scatchard equation (Equation (S8)) [72] and the corresponding Scatchard plots (Figures S7 and S8). Similarly, with the Kq values (Table 4), AuNRs@PEG@fenH and AuNRs@PEG@4′BrfenH have higher K values than free fenH and 4′BrfenH [47], and significantly higher than AuNRs@PEG and AuNRs@PEG@NAP [25]. The herein reported AuNRs present similar or higher SA-affinity than other reported Au nanoparticles [77,78] or nanoclusters [79]. A comparison with the SA-binding constants of recently reported Co(II) complexes with 4′Brfen- ligands (when expressed in (mg/mL)^−1^ instead of M^−1^, Table S1) [47] reveals that AuNRs@PEG@4′BrfenH has higher affinity for both albumins.

The affinity for serum albumins documented above has implications for the behavior of the nanoplatforms in circulation, which were not examined experimentally in the present work. The colloidal stability of the nanoplatforms in the presence of albumin or serum, the kinetics of any aggregation, the formation of a protein corona and the rate of drug release in the presence of albumin were not determined. PEGylation would be expected to limit opsonization and to retard aggregation, and the strong albumin affinity recorded here suggests that albumin could additionally act as a sink for the released drug, but neither expectation was verified in this study. Stability measurements in serum-containing media, together with release experiments performed in the presence of albumin, are required before the behavior of the platform in circulation can be assessed and are identified as future work.

Conclusively, the assignment of an intercalative interaction as the most consistent binding mode is based on the convergence of three independent and well-established lines of evidence: (a) the increase in relative DNA viscosity upon addition of the AuNRs, which is diagnostic of base-pair separation/lengthening characteristic of intercalation; (b) the displacement of the classical intercalator ethidium bromide from the EB–DNA adduct, with static quenching constants consistent with EB replacement; and (c) the temperature-dependent thermodynamic parameters (ΔH > 0, ΔS > 0, ΔG < 0), indicating spontaneous, hydrophobically driven binding stabilized by π–π stacking. It should be mentioned that high-resolution structural confirmation would require additional methods beyond the scope of the present study.

It should be emphasized that these are cell-free measurements performed on isolated CT DNA. They establish the intrinsic affinity and the likely binding mode of the fenamate moiety, but they do not demonstrate that the nanoplatforms are internalized by cells, that they escape the endolysosomal compartment, or that they reach nuclear DNA. No cellular uptake or intracellular localization experiments were performed, and the DNA-binding results are therefore not presented as the mechanism underlying the cytotoxicity reported in Section 3.5.

### 3.5. Evaluation of Cytotoxicity

**Figure 8:**
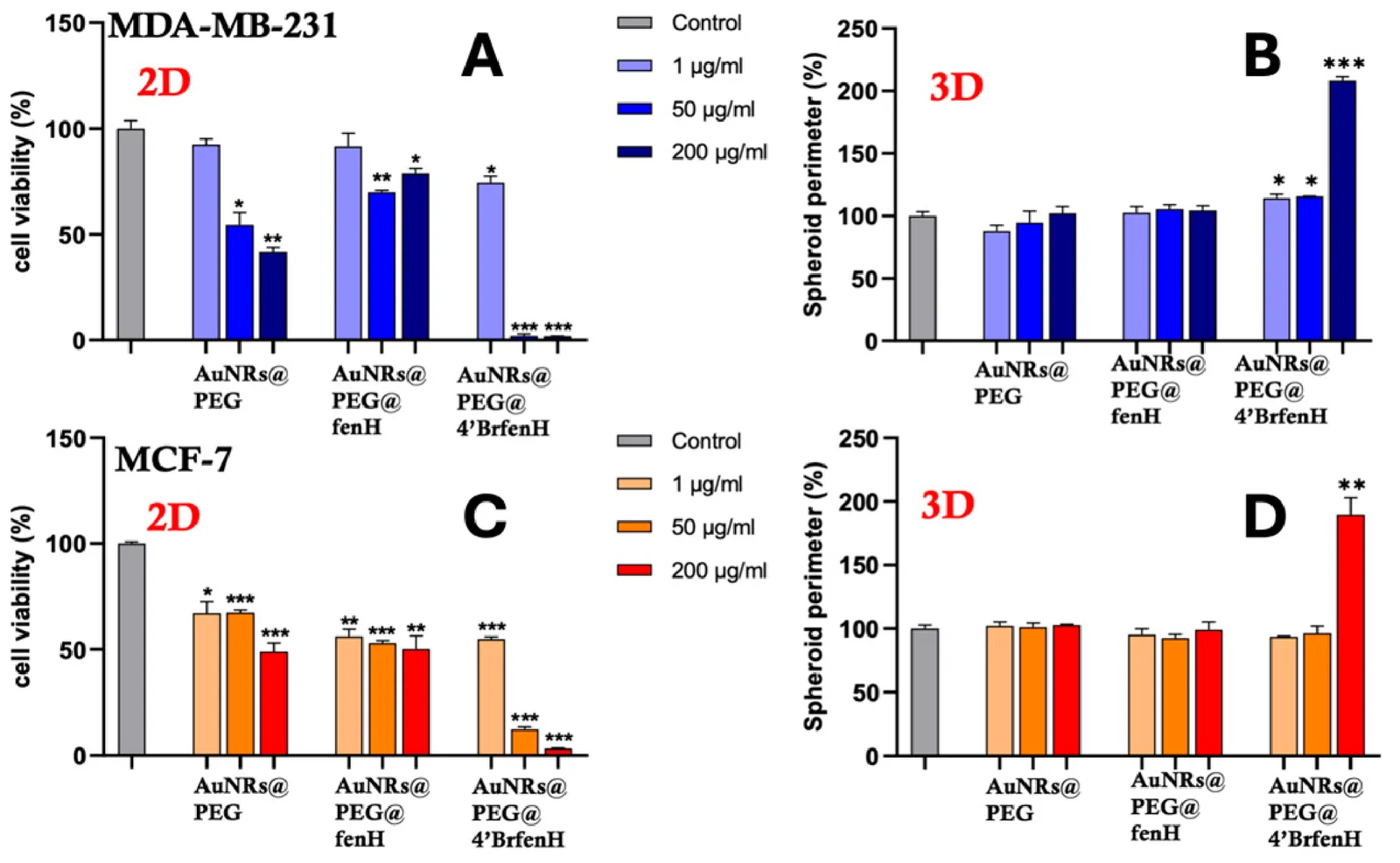
(**A**) Quantification graph of MDA-MB-231 proliferative capability in the presence of AuNRs@PEG, AuNRs@PEG@fenH and AuNRs@PEG@4′BrfenH at 1, 50 and 200 µg/mL. (**B**) Quantification graph of MDA-MB-231 spheroid perimeter in the presence of AuNRs@PEG, AuNRs@PEG@fenH and AuNRs@PEG@4′BrfenH at 1, 50 and 200 µg/mL. (**C**) Quantification graph of MCF-7 proliferative capability in the presence of AuNRs@PEG, AuNRs@PEG@fenH and AuNRs@PEG@4′BrfenH at 1, 50 and 200 µg/mL. (**D**) Quantification graph of MCF-7 spheroid perimeter in the presence of AuNRs@PEG, AuNRs@PEG@fenH and AuNRs@PEG@4′BrfenH at 1, 50 and 200 µg/mL. Each bar represents mean ± SD values. An asterisk (*) indicates statistically significant differences (*p* < 0.05), two asterisks indicate statistically significant differences *p* < 0.01, and three asterisks indicate statistically significant differences (*p* < 0.001), compared to the control samples (untreated cells).

To evaluate the biological effects of AuNRs@PEG, AuNRs@PEG@fenH and AuNRs@PEG@4′BrfenH on the proliferative capacity of the highly aggressive MDA-MB-231 cells and the less metastatic MCF-7 cells, cytotoxicity assays were conducted. Specifically, the AuNRs@PEG, AuNRs@PEG@fenH and AuNRs@PEG@4′BrfenH were applied at concentrations of 1, 50, and 200 µg/mL, and both cell viability and spheroid perimeter were subsequently assessed (Figure 8 and Figures S9–S12).

In 2D cell cultures shown in Figure S9, treatment with 1 µg/mL of both AuNRs@PEG and AuNRs@PEG@fenH resulted in a slight reduction in the viability of MDA-MB-231 cells by approximately 8% (Figure 8A). In contrast, treatment with 1 µg/mL of AuNRs@PEG@4′BrfenH led to a statistically significant reduction of about 25%. At a higher concentration of 50 µg/mL, AuNRs@PEG and AuNRs@PEG@fenH reduced MDA-MB-231 cell viability by approximately 45% and 21%, respectively. Notably, treatment with 50 µg/mL of AuNRs@PEG@4′BrfenH resulted in a pronounced and statistically significant decrease in cell viability, approaching 98%. Furthermore, at a concentration of 200 µg/mL, AuNRs@PEG reduced cell viability by approximately 59%, while AuNRs@PEG@fenH reduced it by 30%. Remarkably, 200 µg/mL AuNRs@PEG@4′BrfenH exhibited the most potent effect, reducing the proliferative capacity of MDA-MB-231 cells by approximately 98% (Figure 8A).

Under 3D culture conditions, MDA-MB-231 spheroids, shown in Figure S10, were either unaffected or only slightly affected by treatment with AuNRs@PEG. In contrast, treatment with both AuNRs@PEG@fenH and AuNRs@PEG@4′BrfenH led to a modest increase in spheroid perimeter across all tested concentrations (Figure 8B). Notably, at a concentration of 200 µg/mL of AuNRs@PEG@4′BrfenH, a pronounced effect was observed, with spheroid perimeter increasing by approximately 108% compared to untreated control spheroids. More precisely, at the structural level, the observed increase in spheroid perimeter can be visually interpreted as a loss of compactness, with the spheroid adopting a loose and less organized architecture (Figure S10).

On the other hand, MCF-7 cells, representing a less aggressive breast cancer cell line, appeared to be more sensitive to all treatments (Figure S11). A statistically significant reduction in cell viability was observed even at the lowest tested concentration (1 µg/mL) for all formulations (Figure 8C). At 50 µg/mL, AuNRs@PEG and AuNRs@PEG@fenH reduced MCF-7 cell viability by approximately 33% and 47%, respectively. The effect was markedly stronger with AuNRs@PEG@4′BrfenH, which decreased viability by around 88%. At 200 µg/mL, all treatments resulted in a general reduction in cell proliferation; however, AuNRs@PEG@4′BrfenH showed the most pronounced impact, reducing MCF-7 cell viability by approximately 97% (Figure 8C).

Like MDA-MB-231, the perimeter of MCF-7 spheroids was either unaffected or only slightly affected, regardless of treatment or concentration (Figure 8D). In contrast, treatment with 200 µg/mL of AuNRs@PEG@4′BrfenH had a statistically significant effect, increasing the spheroid perimeter by approximately 90% compared to the control. In Figure S12, the impact of this treatment is also evident in MCF-7 cells, as a looser structural organization is observed after 24 h of treatment. Notably, the MCF-7 spheroids appear to be structurally affected even more than those of the MDA-MB-231 cell line.

Two limitations of the present cytotoxicity evaluation should be stated explicitly. First, pronounced effects are observed only at relatively high concentrations (≈50 µg/mL in 2D and ≈200 µg/mL in 3D cultures), and no non-malignant cell line was included in the study; the selectivity of the nanoplatforms towards cancer cells has therefore not been established, and the intrinsic activity of the drug-free AuNRs@PEG control at 200 µg/mL noted above makes such a comparison all the more necessary. Second, the platform carries no targeting ligand, so its accumulation would rely upon passive mechanisms alone, and no basis for the selective killing of cancer cells is claimed here. The terminal thiol chemistry of the PEG shell readily permits the conjugation of targeting moieties, and the coupling of such ligands, together with evaluation against non-malignant cells and determination of the corresponding selectivity indices, constitutes a necessary next step before any translational potential can be assessed.

The principal cytotoxicity concern for seed-mediated AuNRs is not the gold core, which is essentially bioinert, but residual cetyltrimethylammonium bromide (CTAB) from the synthesis. Surface passivation through PEGylation displaces CTAB and substantially reduces cytotoxicity. This is consistent with the low intrinsic effect of the AuNRs@PEG control reported in this work and with the near-neutral zeta potential after PEGylation (from +34.8 mV to ~+1.2 mV), and it agrees with established reports that surface chemistry, rather than the gold itself, governs AuNR–cell interactions and toxicity [35]. The PEG layer provides a steric “stealth” coating that improves colloidal stability against aggregation and limits opsonization and protein-corona formation in biological media [34,35,36]. Unlike many organic carriers, the gold core is not biodegradable; after systemic administration, AuNRs are known to accumulate predominantly in the organs of the mononuclear phagocyte system (liver and spleen) and are cleared slowly, which remains a recognized limitation for clinical translation. We have therefore clarified that the present study is an in vitro proof-of-concept and that dedicated in vivo biodistribution, clearance and long-term toxicity studies are required before any translational claim can be made.

The drug-free AuNRs@PEG control also reduced cell viability appreciably at the highest dose (200 µg/mL), and this intrinsic effect should not be disregarded when the loaded systems are evaluated: the activity attributable to the drug is the increment over this control rather than the difference from untreated cells. Two observations indicate substantial removal of CTAB during purification, namely the disappearance of the CTAB CH^2^/CH^3^ bands at 2918 and 2848 cm^−1^ in the FT-IR spectra and the fall of the ζ-potential from +34.8 to ≈+1.2 mV. Residual CTAB was nevertheless not quantified in this work, so trace amounts cannot be excluded, and the residual activity of the carrier is plausibly associated with such traces together with the high particle load; this attribution therefore remains tentative.

## 4. Conclusions

This study explored the interaction of gold nanorods (AuNRs) functionalized with polyethylene glycol (PEG) and loaded with non-steroidal anti-inflammatory drugs (NSAIDs), fenamic acid (fenH), and its bromo-derivative (4′BrfenH), with biomacromolecules and their cytotoxic effects on breast cancer cell lines. The findings demonstrated that both AuNRs@PEG@fenH and AuNRs@PEG@4′BrfenH exhibited significant π–π stacking interactions with calf-thymus (CT) DNA, suggesting the potential intercalation of the fenamic moiety between DNA bases. This interaction was further supported by DNA-viscosity measurements and the ability of the nanorods to displace the reference intercalator ethidium bromide (EB) from the EB–DNA adduct.

The herein studied AuNRs showed a significant affinity for bovine and human serum albumins and may bind tightly to them. The affinity of the AuNR loaded with fenH and 4′BrfeH is much higher than that of AuNRs@PEG. The binding constants of both nanoplatforms (AuNRs@PEG@fenH and AuNRs@PEG@ 4′BrfenH) studied herein to biomacromolecules are comparable with other reported similar systems, including gold nanoparticles and nanoclusters, and previously reported Co(II) complexes of 4′-bromo-fenamic acid.

Cytotoxicity assays in 2D cultures revealed that AuNRs@PEG@4′BrfenH induced the most significant reduction in cell viability in both MDA-MB-231 and MCF-7 cell lines at concentrations of 50 and 200 µg/mL. In 3D cultures, treatments with various concentrations of AuNRs@PEG and AuNRs@PEG@fenH had a slight impact on spheroid perimeter in both cell lines. However, a notable and statistically significant increase in spheroid size was observed following treatment with 200 µg/mL of AuNRs@PEG@4′BrfenH. This increase suggests a loosening of spheroid structure, with both cell lines appearing particularly affected, resulting in a less compact and more disorganized architecture. AuNRs@PEG@4′BrfenH nanoplatform represents a promising in vitro antitumor platform, warranting further in vivo evaluation, especially in tumor contexts where NSAID-based agents have shown anticancer potential. However, further studies incorporating direct comparisons with the corresponding free drug formulations will be necessary to more comprehensively elucidate the therapeutic contribution and translational potential of the developed nanoplatform. Furthermore, future investigations should incorporate 3D spheroid models as primary experimental models since they more accurately recapitulate the structural and physiological complexity of the in vivo tumor microenvironment, thereby enabling a more clinically relevant evaluation of the therapeutic efficacy of the carrier–NSAID combination.

The nanoplatforms released their payload preferentially under alkaline conditions; this behavior is ascribed mainly to the ionization and solubility of the fenamates rather than to a responsive carrier, and matched dissolution controls on the free drugs would be required in order to apportion the two contributions. The DNA- and albumin-binding data are cell-free measurements of intrinsic affinity and do not establish the intracellular mechanism of the observed cytotoxicity. Further work should therefore include comparative free-drug dissolution in the same buffers, ζ-potential and DLS characterization as a function of pH in the release media, quantification of residual CTAB, and cellular uptake and intracellular-localization studies, alongside direct comparison with the free drugs. Evaluation against non-malignant cell lines in order to establish selectivity indices, assessment of colloidal stability and of drug release in serum-containing media, and the conjugation of targeting ligands to the PEG shell are likewise required before any translational potential can be assessed. It should also be noted that the alkaline-biased release profile is not matched to the mildly acidic extracellular microenvironment of solid tumors, and that the present platform is accordingly not proposed as a tumor-microenvironment-responsive delivery system.

## Figures and Tables

**Table 1:** UV-vis spectral features of the interaction of the compounds with CT DNA. UV-band (λ_max_, in nm) (percentage of the observed hyper-/hypo-chromism (ΔA/A_0_, in %), blue-/red-shift in the λ_max_ (Δλ, in nm)) and DNA-binding constants (K_b_, in M^−1^).

Compound	λ_max_ (nm) (ΔA/A_ο_ (%), ^a^ Δλ (nm) ^b^)	K_b_ (M^−1^)
AuNRs@PEG@fenH	287 (+1, ^a^ −2 ^b^); 328 (−11, 0)	2.12 (±0.10) × 10^4^
AuNRs@PEG@4′BrfenH	289 (+3, −2); 335 (−8, 0)	1.15 (±0.02) × 10^5^
fenH	290 (+5, +9); 350 (<−50, elim ^c^)	1.02 (±0.08) × 10^6^
4′BrfenH [47]	301 (+8, +9); 347 (−40, elim)	4.42 (±0.30) × 10^4^
AuNRs@PEG [25]	263 (−1, 0); 525 (−9, +5)	8.84 (±0.45) × 10^4^
AuNRs@PEG@NAP [25]	267 (+10, 0); 319 (−9, +2); 530 (−10, +1)	5.25 (±0.07) × 10^4^

^a^ “+” denotes hyperchromism, “−” denotes hypochromism; ^b^ “+” denotes red–shift, “−” denotes blue–shift; ^c^ “elim” = the band is eliminated.

**Table 2:** Thermodynamic parameters of the compounds for the interaction with CT DNA at three different temperatures.

Compound	T (K)	K_b_ (M^−1^)	ΔG (Kcal M^−1^)	ΔH (Kcal M^−1^)	ΔS (cal M^−1^ K^−1^)
AuNRs@PEG@fenH	295	2.12 (±0.10) × 10^4^	−2.534		
334 nm	302	2.42 (±0.12) × 10^4^	−2.629	+2.176	+15.95
	309	3.13 (±0.16) × 10^4^	−2.758		
AuNRs@PEG@4′BrfenH	295	1.15 (±0.02) × 10^5^	−2.965		
335 nm	302	1.20 (±0.04) × 10^5^	−3.046	+0.671	+2.84
	309	1.30 (±0.25) × 10^5^	−3.137		
AuNRs@PEG [25]	291	8.84 (±0.45) × 10^4^	−2.861		
523 nm	300	3.28 (±0.07) × 10^4^	−2.692	−5.374	−8.74
	310	2.38 (±0.04) × 10^4^	−2.696		
AuNRs@PEG@NAP [25]	291	5.25 (±0.07) × 10^4^	−2.729		
323 nm	300	8.85 (±0.17) × 10^4^	−2.949	+4.618	+25.24
	310	16.20 (±0.18) × 10^4^	−3.209		

**Table 3:** Fluorescence features of the EB-displacement studies: percentage of EB-DNA fluorescence quenching (ΔI/I_0_, %), Stern–Volmer constants (K_SV_) and quenching constants of the EB-DNA fluorescence (K_q_) for the compounds.

Compound	ΔI/I_0_ (%)	K_SV_ ((mg/mL)^−1^)	K_q_ ((mg/mL)^−1^s^−1^)
AuNRs@PEG@fenH	29.5	134.8 ± 2.9	5.86 (±0.13) × 10^9^
AuNRs@PEG@4′BrfenH	21.5	142.8 ± 3.3	6.21 (±1.45) × 10^9^
fenH	46.8	79.1 ± 5.5 1.68 (±0.12) × 10^4 a^	3.44 (±0.24) × 10^9^ 7.32 (±0.05) × 10^11 b^
4′BrfenH [47]	39.1	88.8 ± 2.6 2.59 (±0.07) × 10^4 a^	3.88 (±0.11) × 10^9^ 1.13 (±0.03) × 10^12 b^
AuNRs@PEG [25]	34.0	10.6 ± 0.4	4.59 (±0.18) × 10^8^
AuNRs@PEG@NAP [25]	53.4	17.9 ± 0.3	7.79 (±0.15) × 10^8^

^a^ expressed in M^−1^; ^b^ expressed in M^−1^s^−1^.

**Table 4:** Fluorescence features of the interaction with albumins. Percentage of quenching of the SA-fluorescence (ΔI/I_0_, %), Stern–Volmer constants (K_SV_), SA-quenching constants (K_q_) and SA-binding constants (K) for the compounds.

Compound	ΔI/I_0_ (%)	K_SV_ ((mg/mL)^−1^)	K_q_ ((mg/mL)^−1^s^−1^)	K ((mg/mL)^−1^)
BSA				
AuNRs@PEG@fenH	87.5	3.42 (±0.17) × 10^3^	3.42 (±0.17) × 10^11^	1.58 (±0.04) × 10^3^
AuNRs@PEG@4′BrfenH	86.9	2.27 (±0.13) × 10^3^	2.27 (±0.13) × 10^11^	1.02 (±0.05) × 10^3^
fenH	69.7	7.58 (±0.27) × 10^2^ 1.61 (±0.06) × 10^5 a^	7.58 (±0.27) × 10^10^ 1.61 (±0.06) × 10^13 b^	1.03 (±0.04) × 10^3^ 2.19 (±0.08) × 10^5 a^
4′BrfenH [47]	76.2	5.95 (±0.34) × 10^2^ 1.74 (±0.10) × 10^5 a^	5.95 (±0.34) × 10^10^ 1.74 (±0.10) × 10^13 b^	9.30 (±0.31) × 10^2^ 2.72 (±0.10) × 10^5 a^
AuNRs@PEG [25]	30.8	9.33 ± 0.33	9.33 (±0.33) × 10^8^	10.10 ± 0.65
AuNRs@PEG@NAP [25]	48.9	19.8 ± 0.40	1.98 (±0.04) × 10^9^	18.30 ± 0.69
HSA				
AuNRs@PEG@fenH	82.7	2.95 (±0.05) × 10^3^	2.95 (±0.05) × 10^11^	3.10 (±0.10) × 10^3^
AuNRs@PEG@4′BrfenH	79.2	1.82 (±0.05) × 10^3^	1.82 (±0.05) × 10^11^	1.39 (±0.03) × 10^3^
fenH	62.7	5.75 (±0.50) × 10^2^ 1.23 (±0.11) × 10^5 a^	5.75 (±0.50) × 10^10^ 1.23 (±0.11) × 10^13 b^	1.38 (±0.06) × 10^3^ 2.94 (±0.12) × 10^5 a^
4′BrfenH [47]	70.0	5.21 (±0.26) × 10^2^ 1.52 (±0.07) × 10^5 a^	5.21 (±0.26) × 10^10^ 1.52 (±0.07) × 10^13 b^	9.14 (±0.02) × 10^2^ 2.67 (±0.06) × 10^5 a^
AuNRs@PEG [25]	25.4	7.12 ± 0.27	7.12 (±0.27) × 10^8^	10.25 ± 0.38
AuNRs@PEG@NAP [25]	65.5	38.2 ± 1.3	3.82 (±0.13) × 10^9^	29.49 ± 3.14

^a^ expressed in M^−1^; ^b^ expressed in M^−1^s^−1^.

## Data Availability

The data supporting this article are included in the Supplementary Materials.

## Supplementary Materials

The following supporting information can be downloaded at: https://www.mdpi.com/article/10.3390/molecules31183328/s1. Section S1: Synthesis of fenamic acid (Scheme S1; ^1^H NMR and AT-IR spectra of fenH); Section S2: Drug-release studies (Equation (S1)); Section S3: Interaction studies with CT DNA (Equations (S2)–(S6)); Section S4: Albumin-binding studies (Equations (S7) and (S8)); Table S1: DNA-, BSA- and HSA-binding constants reported for 4′BrfenH and its Co(II) complexes; Figure S1: calibration lines of the emission intensity of fenH and 4′BrfenH; Figure S2: plots of [DNA]/(ε_A_−ε_f_) versus [DNA]; Figure S3: van’t Hoff plots; Figure S4: Stern–Volmer plot of EB-DNA fluorescence; Figures S5 and S6: Stern–Volmer plots of BSA and HSA fluorescence; Figures S7 and S8: Scatchard plots of BSA and HSA; Figures S9 and S11: cell viability of MDA-MB-231 and MCF-7 in 2D cultures; Figures S10 and S12: spheroid compactness of MDA-MB-231 and MCF-7 in 3D cultures. Additional references [45,46,47,63,72,73,75,76] are cited in the Supplementary Materials, where they appear as a separate reference list (refs. 1–8).
