# Supplementary material for: Nanoplatforms of Pegylated Gold Nanorods Loaded with Non-Steroidal Anti-Inflammatory Drugs: Delivery and Biological Evaluation
Source: Molecules. 2026 Sep 19;31(18):3328. doi: 10.3390/molecules31183328 (PMC13609854; doi:10.3390/molecules31183328)
Supplement: Supplementary file 1 [file molecules-31-03328-s001.zip › molecules-4554049-supplementary.pdf]

Nanoplatforms of Pegylated Gold Nanorods Loaded with Non-Steroidal Anti-Inflammatory Drugs: Delivery and Biological Evaluation

Eleni Zygouri [1], Chrisavgi Gourdoupi [2], Spyros Kremmydas [2], Nikos Karamanos [2,*], Georgios Malis [3], George Psomas [3,*], Vlasoula Bekiari [4] and Vassilis Tangoulis [1,*]

[1] Laboratory of Inorganic Chemistry, Department of Chemistry, University of Patras, 26504 Patras, Greece; eleni0503zig@hotmail.com
[2] Biochemistry, Biochemical Analysis & Matrix Pathobiology Research Group, Laboratory of Biochemistry, Department of Chemistry, University of Patras, 26504 Patras, Greece; up1061190@ac.upatras.gr (C.G.)
[3] Department of General and Inorganic Chemistry, Faculty of Chemistry, Aristotle University of Thessaloniki, 54124 Thessaloniki, Greece; georgosmalis@gmail.com
[4] Department of Agriculture, University of Patras, 30200 Messolonghi, Greece; bbekiari@upatras.gr
* Correspondence: n.k.karamanos@upatras.gr (N.K.); gepsomas@chem.auth.gr (G.P.); vtango@upatras.gr (V.T.)

Supplementary material

Index

EXPERIMENTAL PROTOCOLS *3*

S1 Synthesis of fenamic acid 3

S2 Drug release studies 5

S3 Interaction studies with CT DNA 6

S3.1 Binding study with CT DNA by UV-vis spectroscopy 6

S3.2 Determination of the thermodynamic parameters 6

S3.3 Viscosity measurements 7

S3.4 EB-displacement studies 7

S4 Albumin-binding studies 8

TABLES *9*

Table S1. DNA-binding constants (K_b, in M^{-1}), BSA-binding constants ($K_{(BSA)}$, in $(mg/mL)^{-1}$ and in M^{-1}) and HSA-binding constants ($K_{(HSA)}$, in $(mg/mL)^{-1}$ and in M^{-1}) reported for 4'BrfenH and its Co(II) complexes. 9

REFERENCES *10*

FIGURES *11*

Figure S1. Calibration lines of the emission intensity of fenH at 460 nm and 4'BrfenH (at 415 nm) as a function of the concentration. 11

Figure S2. Plot of [DNA]/(ε_A-ε_f) *versus* [DNA] for AuNRs@PEG@fenH and AuNRs@PEG@4'BrfenH. 11

Figure S3. van't Hoff plots for the interaction of CT DNA with AuNRs@PEG@fenH and AuNRs@PEG@4'BrfenH. 12

Figure S4. Stern-Volmer quenching plot of EB-DNA fluorescence for AuNRs@PEG@fenH and AuNRs@PEG@4'BrfenH. 12

Figure S5. Stern-Volmer quenching plot of BSA fluorescence for AuNRs@PEG@fenH and AuNRs@PEG@4'BrfenH. 13

Figure S6. Stern-Volmer quenching plot of HSA fluorescence for AuNRs@PEG@fenH and AuNRs@PEG@4'BrfenH. 13

Figure S7. Scatchard plot of BSA for AuNRs@PEG@fenH and AuNRs@PEG@4'BrfenH. 14

Figure S8. Scatchard plot of HSA for AuNRs@PEG@fenH and AuNRs@PEG@4'BrfenH. 14

Figure S9. Cell viability of MDA-MB-231 in 2D cell cultures in the presence of AuNRs@PEG, AuNRs@PEG@fenH and AuNRs@PEG@4'BrfenH at 1, 50 and 200 μg/mL. Representative images of MDA-MB-231 after 0 and 24 h of treatment. 15

Figure S10. Spheroids compactness of MDA-MB-231 in 3D cultures. Representative images of MDA-MB-231 spheroids in the presence of AuNRs@PEG, AuNRs@PEG@fenH and AuNRs@PEG@4'BrfenH at 1, 50 and 200 μg/mL after 0 and 24 h of treatment. 17

Figure S11. Cell viability of MCF-7 in 2D cell cultures in the presence of AuNRs@PEG, AuNRs@PEG@fenH and AuNRs@PEG@4'BrfenH at 1, 50 and 200 μg/mL. Representative images of MCF-7 after 0 and 24 h of treatment. 19

Figure S12. Spheroids compactness of MCF-7 in 3D cultures. Representative images of MCF-7 spheroids in the presence of AuNRs@PEG, AuNRs@PEG@fenH and AuNRs@PEG@4'BrfenH at 1, 50 and 200 μg/mL after 0 and 24 h of treatment. 21

EXPERIMENTAL PROTOCOLS

S1 Synthesis of fenamic acid

2-chlorobenzoic acid, aniline, K_2CO_3 and a catalytic amount of $CuSO_4$ (Scheme S1) were dissolved in N,N-dimethylformamide in a 1:2:0.5 ratio and heated under reflux at 153 °C for 24 h. The precipitate, after acidification of the reaction and stirring for 24 h, was recrystallized to give the pure compound. The resultant compound was characterized with ^1H NMR and IR spectroscopies and the obtained ^1H NMR spectral data are in correlation with the literature [1,2].

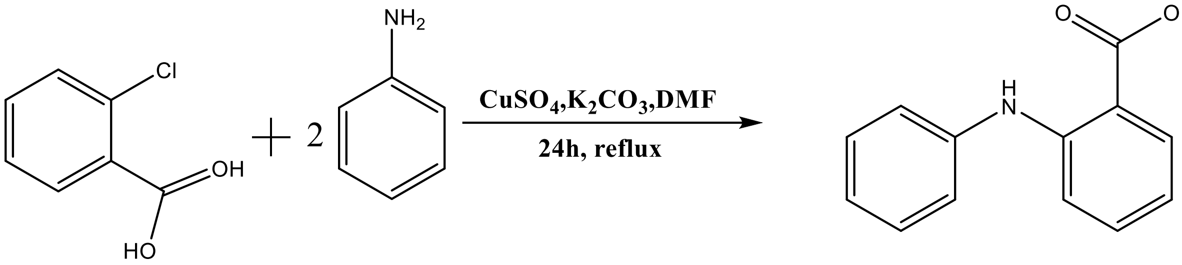

Scheme S1. Synthesis of fenamic acid using 2-chlorobenzoic acid (1 eq), aniline (2 eq), $CuSO_4$ (catalytic), K_2CO_3 (0.5 eq), DMF, reflux, 24 h, 85% yield.

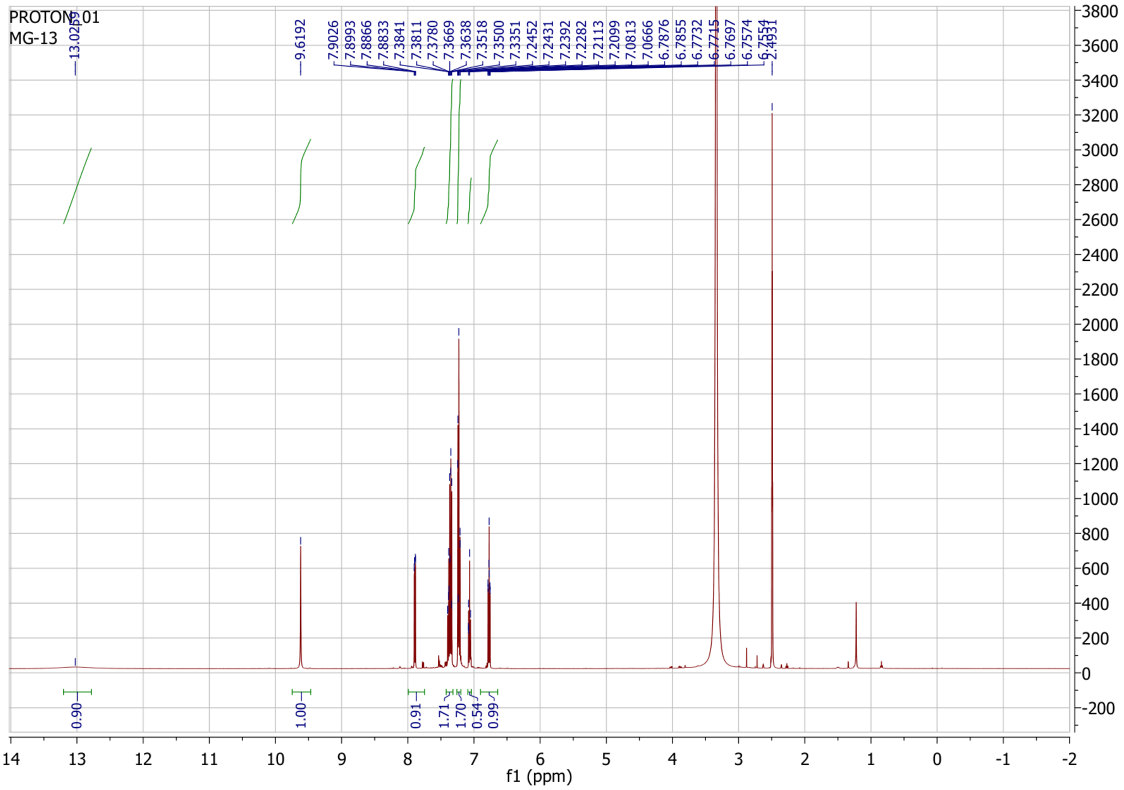

^1H NMR spectrum of fenH in DMSO-d_6.

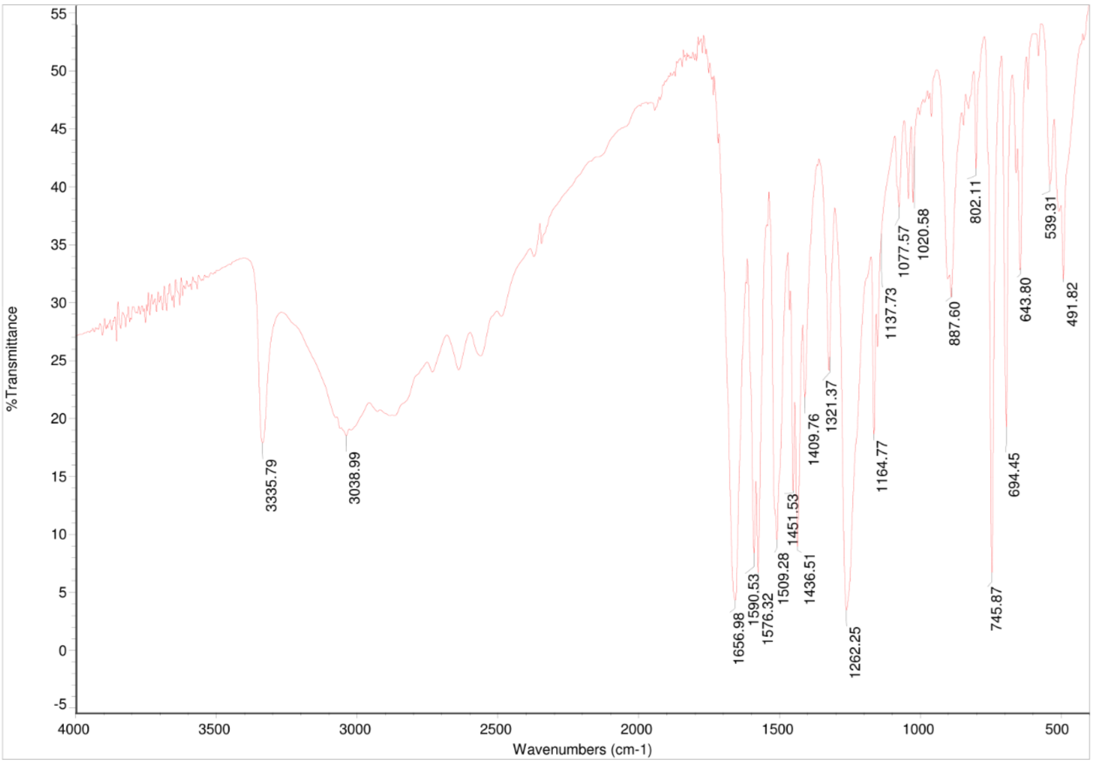

AT-IR spectrum of fenH.

S2 Drug release studies

The fluorescence (excitation and emission) spectra of the AuNRs (AuNRs@PEG@fenH and AuNRs@PEG@4’BrfenH) in aqueous suspensions at a nanoplatform concentration of 0.1 g/L were recorded on a Shimadzu RF 5301PC emission spectrophotometer. This concentration was selected so as to afford a suitable signal level for the representative spectra reproduced in Figure 4 (left) of the main text; the loading was determined separately, on a dispersion containing 0.5 g/L of nanoplatform and 0.04 g/L of drug, from which a loading capacity of ≈80 mg/g follows.

The drug-release behavior fenH and 4’BrfenH was investigated according to the following procedure: 5 mg of AuNRs@PEG@fenH or AuNRs@PEG@4’BrfenH were dispersed in 10 mL of each of three buffered solutions (pH = 4.1, 6.8 and 10.1) under stirring at room temperature. The buffered media were: pH 4.1, potassium hydrogen phthalate/phthalate (0.1 M); pH 6.8, NaH_2PO_4/Na_2HPO_4 (0.1 M); pH 10.1, $NaHCO_3/Na_2CO_3$ (0.1 M). The three media are of comparable ionic strength, in the range of approximately 0.10–0.15 M.
Quantification relies on the strong quenching of the surface-bound drug emission by the gold core; release into the medium restores the fluorescence, the recovered intensity being proportional to the released amount as determined from the calibration curves.

At predetermined time intervals, the solution was transferred into a cuvette and the emission intensity of fenH at 460 nm or 4’BrfenH at 415 nm was recorded. The cumulative release (%) was expressed as:

$$Cumulative\ release\ (\%) = \frac{W_t}{W_{drug}} \times 100 \qquad \text{(equation S1)}$$

where W_t and W_{drug} indicate the quantity of drug released from the AuNRs at time t, and the total amount of loaded drug, respectively. W_t and W_{drug} were calculated from the calibration curve of free fenH and 4’BrfenH (emission intensity at 460 nm and 415 nm, respectively, for various concentrations).

The calibration lines of the emission intensity of fenH at 460 nm and 4’BrfenH at 415 nm were formed as a function of concentration. Because fenH and 4′BrfenH are practically insoluble in water, the calibration standards were prepared as follows. A stock solution of fenH or 4′BrfenH was prepared in absolute ethanol at a concentration of 10 mg/mL. Standard solutions of 0.005, 0.01, 0.05 and 0.1 mg/mL were then prepared in a final volume of 50 mL by appropriate dilution of the stock solution with the corresponding buffered aqueous medium: aliquots of 25, 50, 250 and 500 µL of the stock solution were transferred to separate 50 mL volumetric flasks, followed by the addition of 475, 450, 250 and 0 µL of absolute ethanol, respectively, and the flasks were then made up to volume with the buffered medium. In this way, the final ethanol content was maintained constant at 1.0 % (v/v) in

all the experimental solutions. The same procedure was applied for all three buffers. The release samples themselves contained no added ethanol.

The release experiments were carried out under non-sink conditions at room temperature over 120 min, in triplicate, and the quantity of drug was determined from calibration curves of the free drug constructed in the corresponding buffer. Because the free drugs were not subjected to matched dissolution measurements in the same media, the fraction of the observed pH dependence arising from drug solubility, as distinct from release from the carrier, could not be determined; the release data are accordingly presented as relative, comparative trends between the two drugs under identical conditions. Values are reported as mean ± SD (n = 3).

S3 Interaction studies with CT DNA

The interaction of the AuNRs with CT DNA was studied by UV-vis spectroscopy at room temperature and variable temperatures, and via competitive studies with EB by fluorescence emission spectroscopy.

S3.1 Binding study with CT DNA by UV-vis spectroscopy

The interaction of the AuNRs with CT DNA was studied by UV-vis spectroscopy in order to investigate the possible binding modes to CT DNA and to calculate the DNA-binding constants (K_b). The K_b constants (in M^{-1}) of the AuNRs were determined by the Wolfe-Shimer equation (eq. S1) [3] and the plots [DNA]/(ε_A-ε_f) versus [DNA] using the UV-vis spectra of the AuNRs (0.01 mg/mL) recorded in the presence of increasing concentrations of CT DNA. According to the Wolfe-Shimer equation (equation S2):

$$\frac{[DNA]}{(\varepsilon_A - \varepsilon_f)} = \frac{[DNA]}{(\varepsilon_b - \varepsilon_f)} + \frac{1}{K_b(\varepsilon_b - \varepsilon_f)} \qquad \text{(equation S2)}$$

where [DNA] is the concentration of DNA in base pairs, ε_A = A_{obsd}/[compound], ε_f = the extinction coefficient for the free AuNR, and ε_b = the extinction coefficient for the AuNR in the fully bound form. K_b is given by the ratio of slope to the y-intercept in plots [DNA]/(ε_A-ε_f) versus [DNA].

S3.2 Determination of the thermodynamic parameters

In order to determine the thermodynamics parameters enthalpy change (ΔH), entropy change (ΔS) and ΔG, the DNA-binding constants of the AuNRs were determined for three different temperatures (291 K, 300 K and 310 K) as described in section S2.1 with equation S2 and the corresponding plots.

The enthalpy change (ΔH) and the entropy change (ΔS) can be calculated from the van't Hoff equation (equation S3):

$$\ln{(K_b)} = -\frac{\Delta \mathrm{H}}{RT} + \frac{\Delta S}{R} \qquad \text{(equation S3)}$$

where ΔH and ΔS can be determined from the plot of ln(K_b) versus (1/T), where – ΔH/R is the slope of the fitting line and ΔS/R is the intercept (R is the universal gas constant). In addition, ΔG may be obtained from the Gibbs–Helmholtz equation (equation S4):

$$\Delta G = \Delta H - T \cdot \Delta S \qquad \text{(equation S4)}$$

S3.3 Viscosity measurements

The viscosity of DNA ([DNA] = 0.1 mM) in buffer solution (150 mM NaCl and 15 mM trisodium citrate at pH 7.0) was measured in the presence of increasing amounts of the AuNRs (up to a concentration of 3.2 mg/L). All measurements were performed at room temperature. The obtained data are presented as $(\eta/\eta_0)^{1/3}$ versus the concentration of the AuNRs, where η is the viscosity of DNA in the presence of the compound, and η_0 is the viscosity of DNA alone in buffer solution.

S3.4 EB-displacement studies

The competition of the AuNRs with EB was investigated by fluorescence emission spectroscopy in order to examine whether the AuNRs may displace EB from its DNA-EB adduct. The CT DNA-EB adduct was formed by pre-treating 20 μM EB and 26 μM CT DNA in buffer (150 mM NaCl and 15 mM trisodium citrate at pH = 7.0). The possible displacement of EB by the AuNRs and subsequently their intercalating effect was studied by the stepwise addition of a certain amount of the solution of each AuNR into the solution of the CT DNA-EB adduct. The solutions were excited at 540 nm and the emission was monitored from 550 to 700 nm with λ_{max}= 592-595 nm and the effect of the addition of each AuNR to the CT-DNA EB solution was recorded. The AuNRs did not display any fluorescence emission bands at room temperature in solution or in the presence of CT DNA or EB under the same experimental conditions ($\lambda_{excitation}$ = 540 nm); therefore, the observed quenching of the EB-DNA solution may be attributed to the displacement of EB from its EB-DNA adduct.

The Stern-Volmer constants (K_{SV}, in $(mg/mL)^{-1}$) were calculated according to the linear Stern-Volmer equation (equation S5) [4] and the respective plots Io/I versus [AuNR].

$$\frac{Io}{I} = 1 + K_q \tau_0 [Q] = 1 + K_{SV}[Q] \qquad \text{(equation S5)}$$

where Io and I are the emission intensities of the EB-DNA solution in the absence and the presence of the AuNRs, respectively, τ_o= the average lifetime of the emitting system without the quencher and K_q = the quenching constant. Taking τ_o = 23 ns as the fluorescence lifetime of the EB-DNA adduct

[5], the quenching constants (K_q, in $(mg/mL)^{-1}s^{-1}$) of the AuNRs were calculated according to equation S5 [4]:

$$K_{SV} = K_q \tau_o \qquad \text{(equation S6)}$$

S4 Albumin-binding studies

In order to investigate if the AuNRs can bind to carrier proteins like serum albumins, we carried out an albumin-binding study by tryptophan fluorescence quenching experiments using bovine serum albumin (BSA, 3 μM) or human serum albumin (HSA, 3 μM) in buffer (containing 15 mM trisodium citrate and 150 mM NaCl at pH 7.0). The quenching of the emission intensity of tryptophan residues at 345 nm for BSA or at 340 nm for HSA was monitored using the AuNRs as quenchers with increasing concentration [4]. The fluorescence emission spectra of the AuNRs were also recorded with λ_{ex} = 295 nm. No fluorescence emission band was recorded, and thus it was not related to the AuNRs. The influence of the inner-filter effect [6] on the measurements was evaluated by equation S6.

$$I_{corr} = I_{meas} \times 10^{\frac{\varepsilon(\lambda_{exc})cd}{2}} \times 10^{\frac{\varepsilon(\lambda_{em})cd}{2}} \qquad \text{(equation S7)}$$

where I_{corr} = corrected intensity, I_{meas} = the measured intensity, c = the concentration of the quencher, d = the cuvette (1 cm), $\varepsilon_{(\lambda exc)}$ and $\varepsilon_{(\lambda em)}$ = the ε of the quencher at the excitation and the emission wavelength, respectively, as calculated from the UV-vis spectra of the AuNRs [6].

The Stern–Volmer and Scatchard graphs were used to study the interaction of the compounds with serum albumins. According to Stern-Volmer quenching equation (equation S5), where Io = the initial tryptophan fluorescence intensity of SA, I = the tryptophan fluorescence intensity of SA after the addition of the quencher, K_q = the SA-quenching constant, K_{SV} = the Stern-Volmer constant, τ_o = the average lifetime of SA without the quencher, and, taking as fluorescence lifetime (τ_o) of tryptophan in SA at around 10^{-8} s [4], K_{SV} can be obtained by the slope of the diagram Io/I versus [AuNR] (Stern-Volmer plots), and subsequently the quenching (K_q) is calculated with equation S6.

From the Scatchard equation (equation S8):

$$\frac{\Delta I / Io}{[Q]} = nK - K\frac{\Delta I}{Io} \qquad \text{(equation S8)}$$

where n is the number of binding sites per albumin and K is the SA-binding constant (in $(mg/mL)^{-1}$) is calculated from the slope in plots (ΔI/Io)/[AuNR] versus (ΔI/Io) and n is given by the ratio of y intercept to the slope [4,7].

TABLES

Table S1. DNA-binding constants (K_b, in M^{-1}), BSA-binding constants ($K_{(BSA)}$, in $(mg/mL)^{-1}$ and in M^{-1}) and HSA-binding constants ($K_{(HSA)}$, in $(mg/mL)^{-1}$ and in M^{-1}) reported for 4’BrfenH and its Co(II) complexes.

**Compound**	**K_b (M^{-1})**	**$K_{(BSA)}$ ($(mg/mL)^{-1}$)**	**$K_{(HSA)}$ ($(mg/mL)^{-1}$)**
4’BrfenH [8]	4.42(±0.30)×10^4	9.30(±0.31)×10^2	* 9.14(±0.02)×10^2
		* 2.72(±0.10)×10^5	2.67(±0.06)×10^5
[Co(4’Brfen)$_2$(MeOH)$_4$] [8]	8.90(±0.19)×10^5	4.22(±0.14)×10^2	6.21(±0.19)×10^2
		* 3.24(±0.11)×10^5	* 4.77(±0.15)×10^5
[Co(4’Brfen)$_2$(phen)(MeOH)$_2$] [8]	6.73(±0.20)×10^5	3.91(±0.14)×10^2	4.08(±0.11)×10^2
		* 3.45(±0.12)×10^5	* 3.60(±0.10)×10^5
[Co(4’Brfen)$_2$(neoc)(MeOH)$_2$] [8]	7.92(±0.75)×10^5	3.29(±0.09)×10^2	4.20(±0.10)×10^2
		* 3.00(±0.08)×10^5	* 3.83(±0.09)×10^5
[Co(4’Brfen)$_2$(bipyam)(MeOH)$_2$] [8]	9.92(±0.14)×10^5	4.39(±0.09)×10^2	4.34(±0.11)×10^2
		* 3.84(±0.08)×10^5	* 3.80(±0.10)×10^5
[Co(4’Brfen)$_2$(Himi)$_2$(MeOH)$_2$] [8]	3.61(±0.10)×10^5	4.03(±0.12)×10^2	3.46(±0.13)×10^2
		* 3.38(±0.10)×10^5	* 2.90(±0.11)×10^5
[Co(4’Brfen)$_2$(py)$_2$(MeOH)$_2$] [8]	2.00(±0.07)×10^6	4.58(±0.16)×10^2	6.79(±0.25)×10^2
		* 3.95(±0.14)×10^5	* 5.82(±0.23)×10^5
[Co(4’Brfen)$_2$(αpic)$_2$(MeOH)$_2$] [8]	1.56(±0.07)×10^6	5.62(±0.17)×10^2	8.03(±0.17)×10^2
		* 5.00(±0.15)×10^5	* 7.15(±0.15)×10^5
[Co(4’Brfen)$_2$(βpic)$_2$(MeOH)$_2$] [8]	1.21(±0.19)×10^6	5.27(±0.15)×10^2	7.02(±0.20)×10^2
		* 4.69(±0.13)×10^5	* 6.20(±0.18)×10^5
[Co(4’Brfen)$_2$(γpic)$_2$(MeOH)$_2$] [8]	9.37(±0.65)×10^5	4.18(±0.09)×10^2	6.27(±0.17)×10^2
		* 3.72(±0.84)×10^5	* 5.58(±0.15)×10^5

* $K_{(BSA/HSA)}$ values expressed in M^{-1}

REFERENCES

[1] A. Martín, R.F. Pellón, M. Mesa, M.L. Docampo, V. Gómez, Microwave-assisted synthesis of N-phenylanthranilic acids in water, Jorunal of Chemical Research 2005 (2005) 561–563. https://doi.org/10.3184/030823405774308998.

[2] M.R. Haider, K. Ahmad, N. Siddiqui, Z. Ali, M.J. Akhtar, N. Fuloria, S. Fuloria, M. Ravichandran, M.S. Yar, Novel 9-(2-(1-arylethylidene)hydrazinyl)acridine derivatives: Target Topoisomerase 1 and growth inhibition of HeLa cancer cells, Bioorg. Chem. 88 (2019) 102962. https://doi.org/10.1016/j.bioorg.2019.102962.

[3] A. Wolfe, G.H. Shimer, T. Meehan, Polycyclic Aromatic Hydrocarbons Physically Intercalate into Duplex Regions of Denatured DNA, Biochemistry 26 (1987) 6392–6396. https://doi.org/10.1021/bi00394a013.

[4] J.R. Lakowicz, Principles of Fluorescence Spectroscopy, Springer US, Boston, MA, 2006. https://doi.org/10.1007/978-0-387-46312-4.

[5] D.P. Heller, C.L. Greenstock, Fluorescence lifetime analysis of DNA intercalated ethidium bromide and quenching by free dye, Biophys. Chem. 50 (1994) 305–312. https://doi.org/10.1016/0301-4622(93)E0101-A.

[6] L. Stella, A.L. Capodilupo, M. Bietti, A reassessment of the association between azulene and [60]fullerene. Possible pitfalls in the determination of binding constants through fluorescence spectroscopy, Chemical Communications (2008) 4744–4746. https://doi.org/10.1039/B808357F.

[7] Y.-Q. Wang, H.-M. Zhang, G.-C. Zhang, W.-H. Tao, S.-H. Tang, Interaction of the flavonoid hesperidin with bovine serum albumin: A fluorescence quenching study, J. Lumin. 126 (2007) 211–218. https://doi.org/10.1016/J.JLUMIN.2006.06.013.

[8] G. Malis, C.N. Banti, A. Tialiou, M.R. Reithofer, A.G. Hatzidimitriou, S.K. Hadjikakou, K.C. Fylaktakidou, G. Psomas, Cobalt(II) Complexes of 4′-Bromo-Fenamic Acid: Antioxidant Properties, Antibacterial Activity, and Interaction with DNA and Albumins, Int. J. Mol. Sci. 26 (2025) 9787. https://doi.org/10.3390/ijms26199787.

FIGURES

Figure S1. Calibration lines of the emission intensity of fenH at 460 nm and 4’BrfenH (at 415 nm) as a function of the concentration.

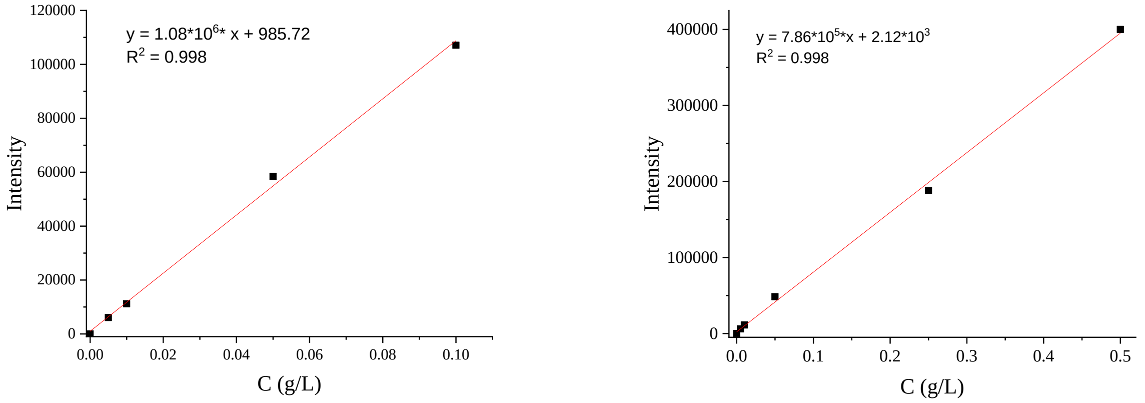

fenH (460 nm) 4’BrfenH (415 nm)

Figure S2. Plot of [DNA]/(ε_A-ε_f) *versus* [DNA] for AuNRs@PEG@fenH and AuNRs@PEG@4’BrfenH.

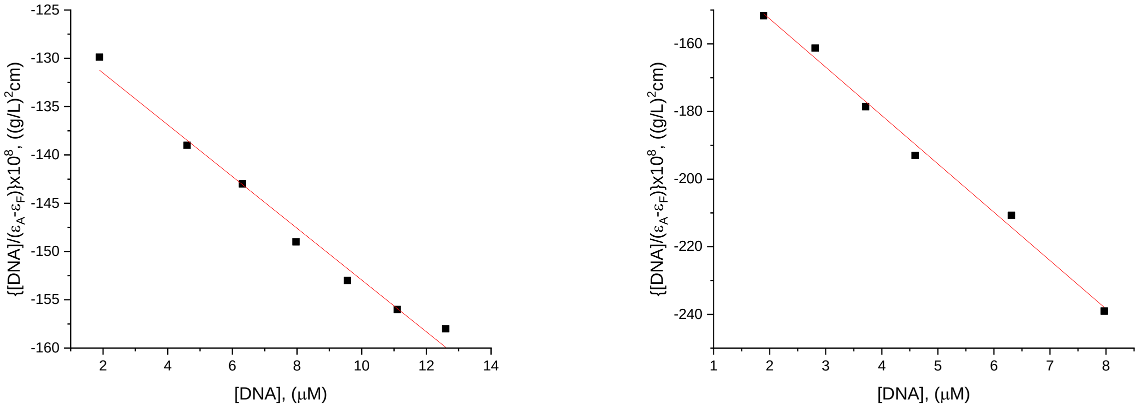

AuNRs@PEG@fenH AuNRs@PEG@4’BrfenH

Figure S3. van't Hoff plots for the interaction of CT DNA with AuNRs@PEG@fenH and AuNRs@PEG@4'BrfenH.

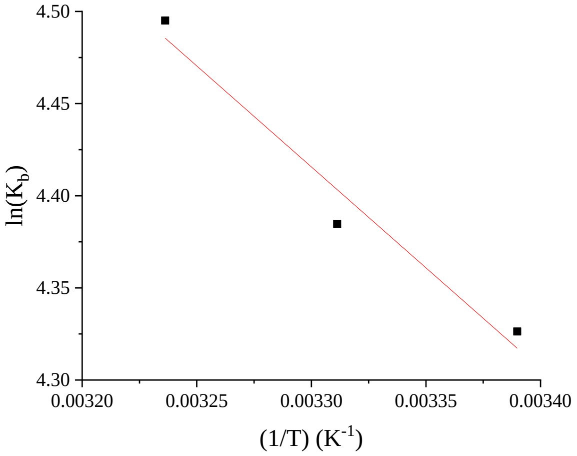

AuNRs@PEG@fenH

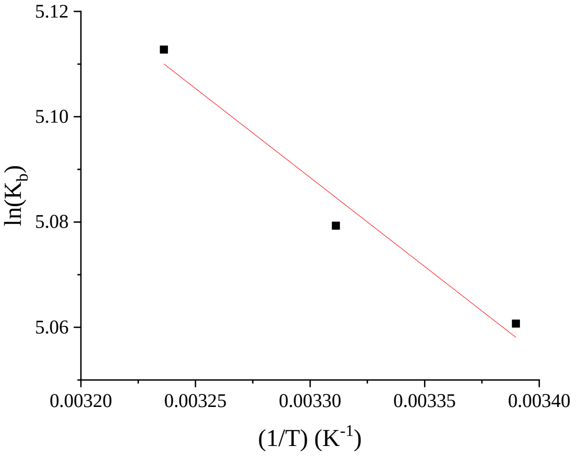

AuNRs @PEG@4'BrfenH

Figure S4. Stern-Volmer quenching plot of EB-DNA fluorescence for AuNRs@PEG@fenH and AuNRs@PEG@4'BrfenH.

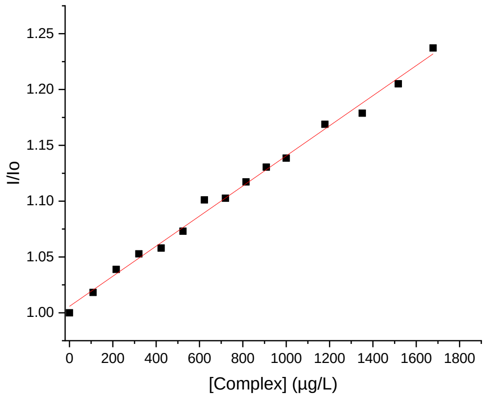

AuNRs@PEG@fenH

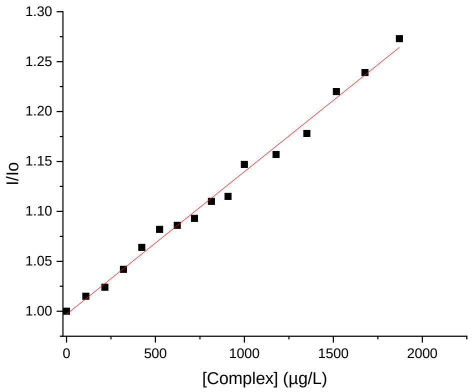

AuNRs@PEG@4'BrfenH

Figure S5. Stern-Volmer quenching plot of BSA fluorescence for AuNRs@PEG@fenH and AuNRs@PEG@4'BrfenH.

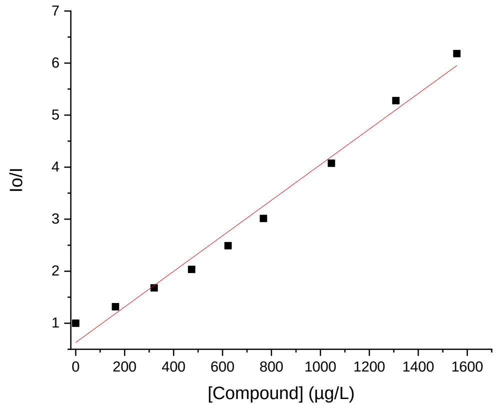

AuNRs@PEG@fenH

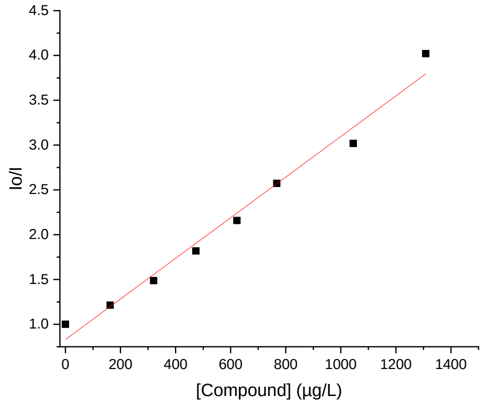

AuNRs@PEG@4'BrfenH

Figure S6. Stern-Volmer quenching plot of HSA fluorescence for AuNRs@PEG@fenH and AuNRs@PEG@4'BrfenH**.**

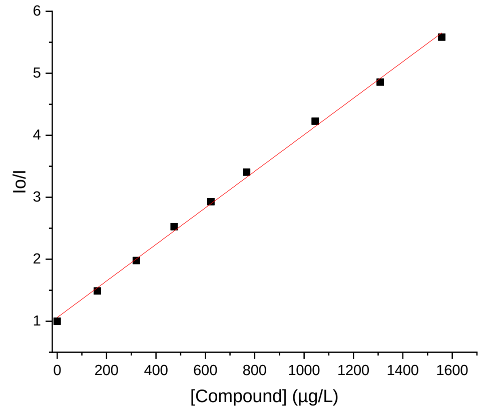

AuNRs@PEG@fenH

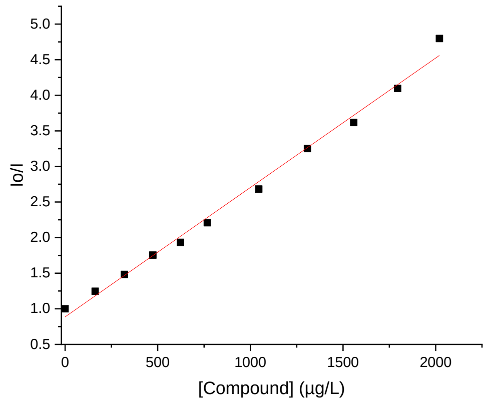

AuNRs@PEG@4'BrfenH

Figure S7. Scatchard plot of BSA for AuNRs@PEG@fenH and AuNRs@PEG@4’BrfenH**.**

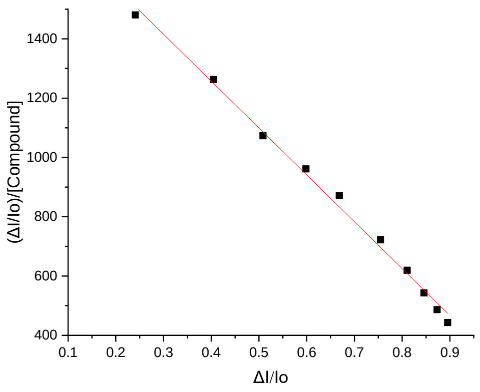

AuNRs@PEG@fenH

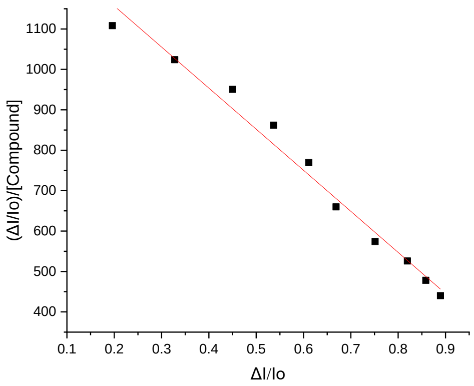

AuNRs@PEG@4’BrfenH

Figure S8. Scatchard plot of HSA for AuNRs@PEG@fenH and AuNRs@PEG@4’BrfenH.

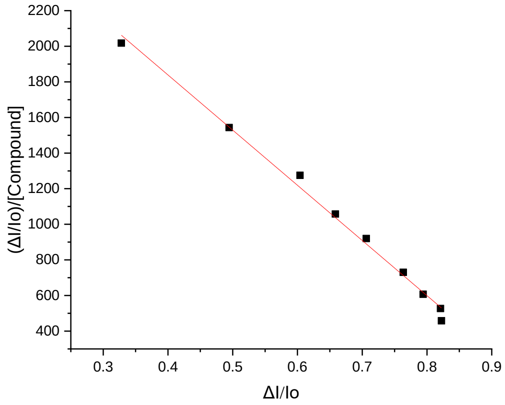

AuNRs@PEG@fenH

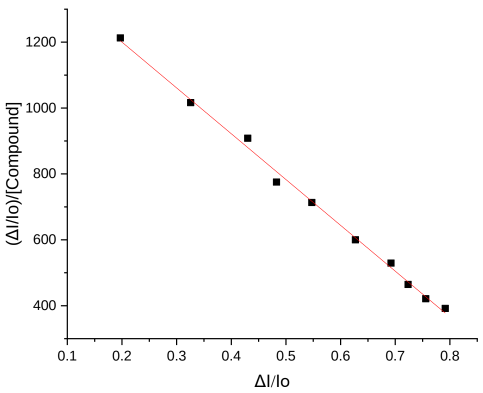

AuNRs@PEG@4’BrfenH

Figure S9. Cell viability of MDA-MB-231 in 2D cell cultures in the presence of AuNRs@PEG, AuNRs@PEG@fenH and AuNRs@PEG@4'BrfenH at 1, 50 and 200 µg/mL. Representative images of MDA-MB-231 after 0 and 24 h of treatment.

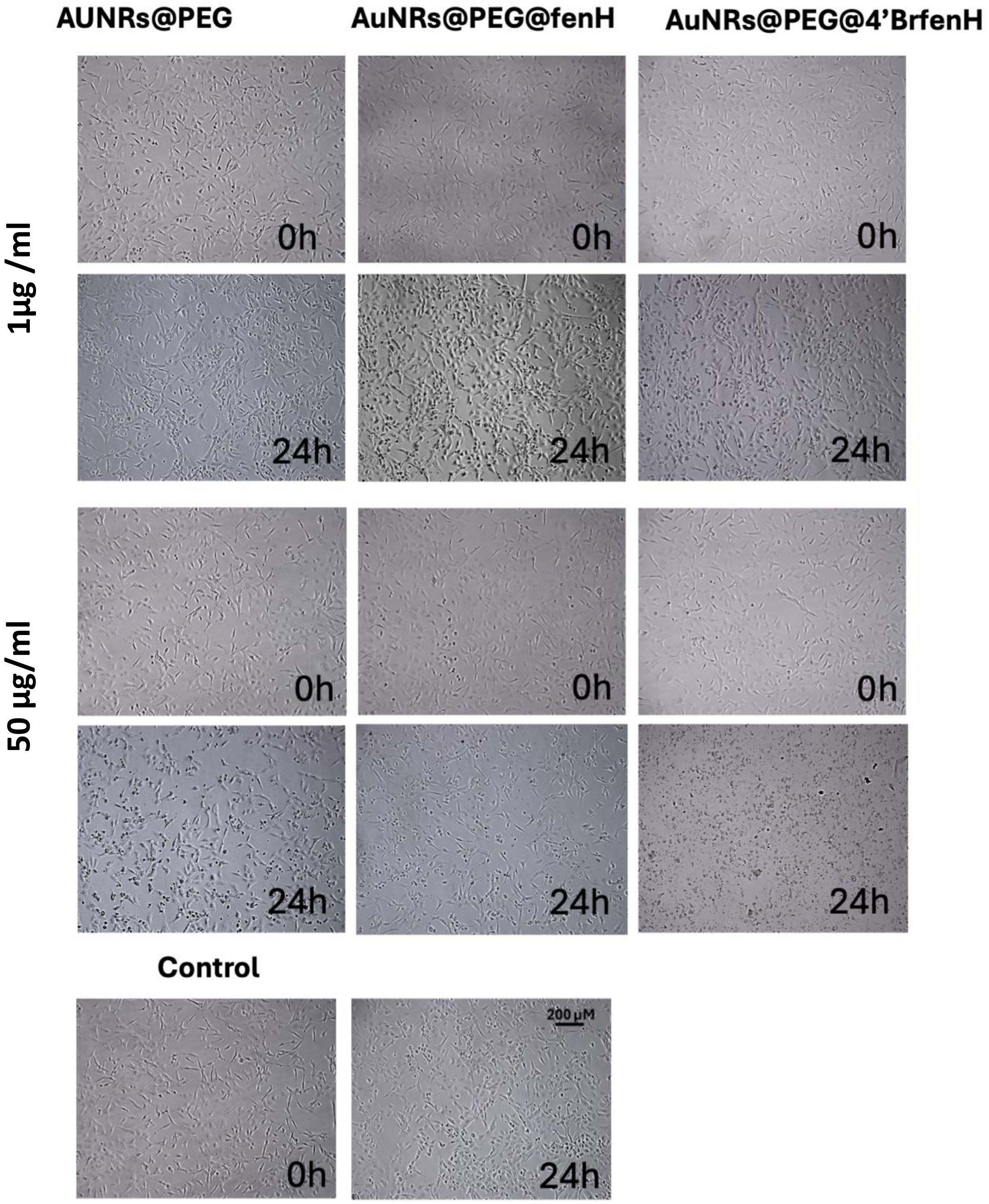

(continued)

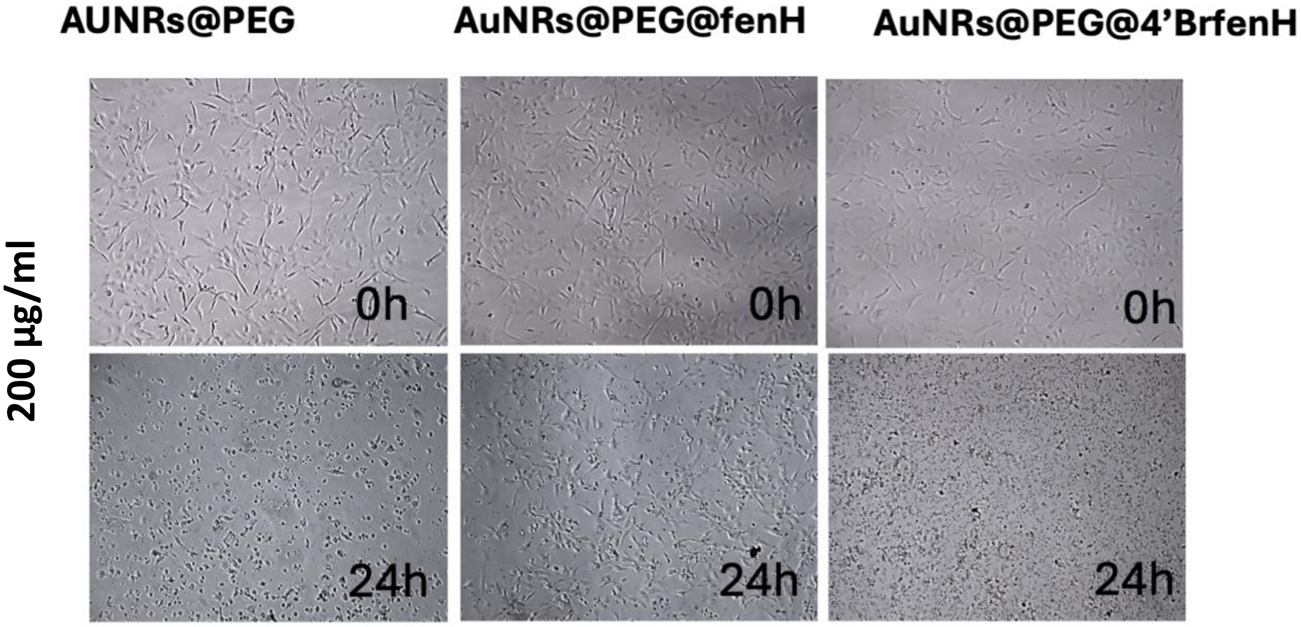
AUNRs@PEG
AuNRs@PEG@fenH
AuNRs@PEG@4'BrfenH
200 µg/ml
0h
0h
0h
24h
24h
24h

Figure S10. Spheroids compactness of MDA-MB-231 in 3D cultures. Representative images of MDA-MB-231 spheroids in the presence of AuNRs@PEG, AuNRs@PEG@fenH and AuNRs@PEG@4’BrfenH at 1, 50 and 200 µg/mL after 0 and 24 h of treatment.

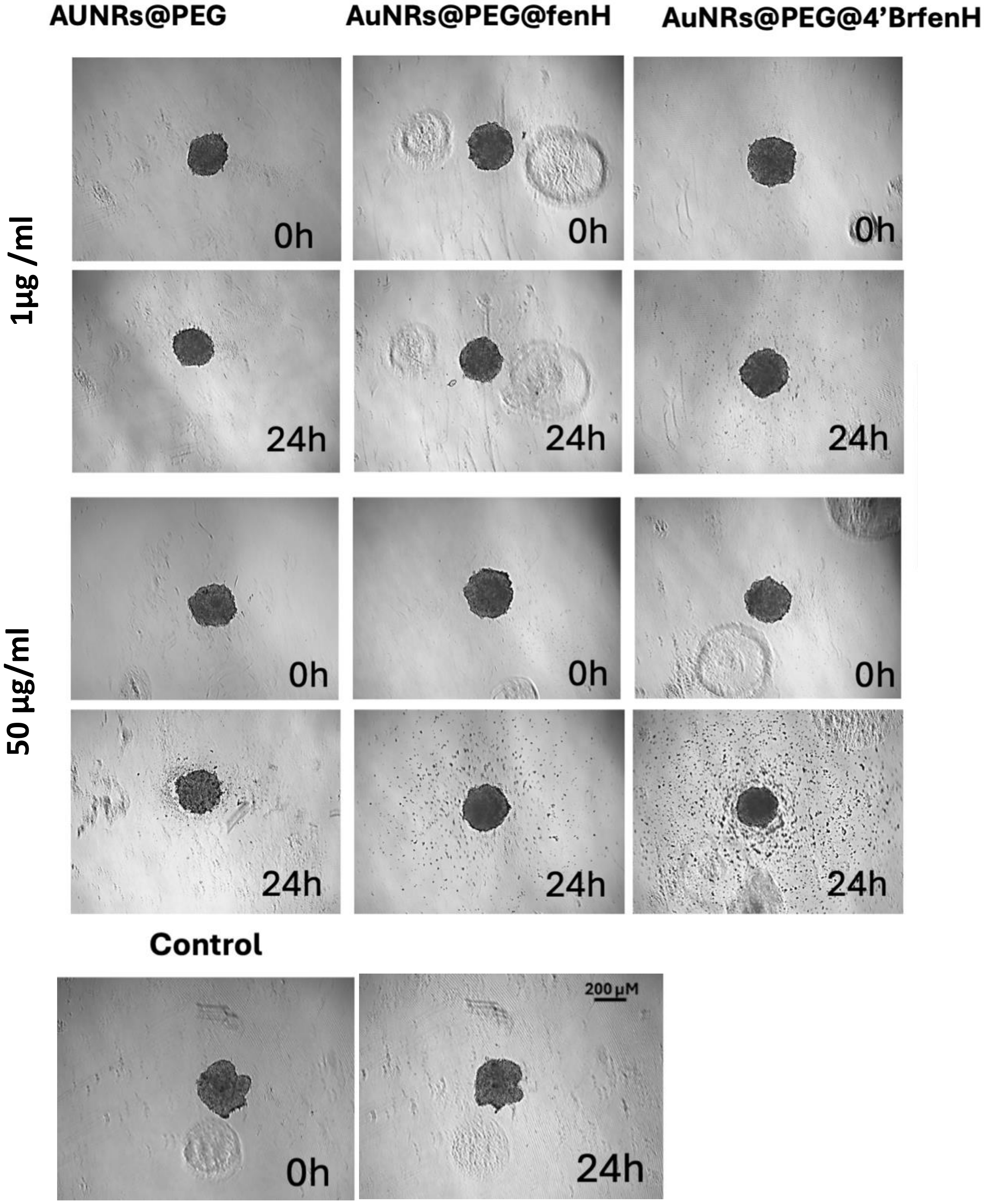

(continued)

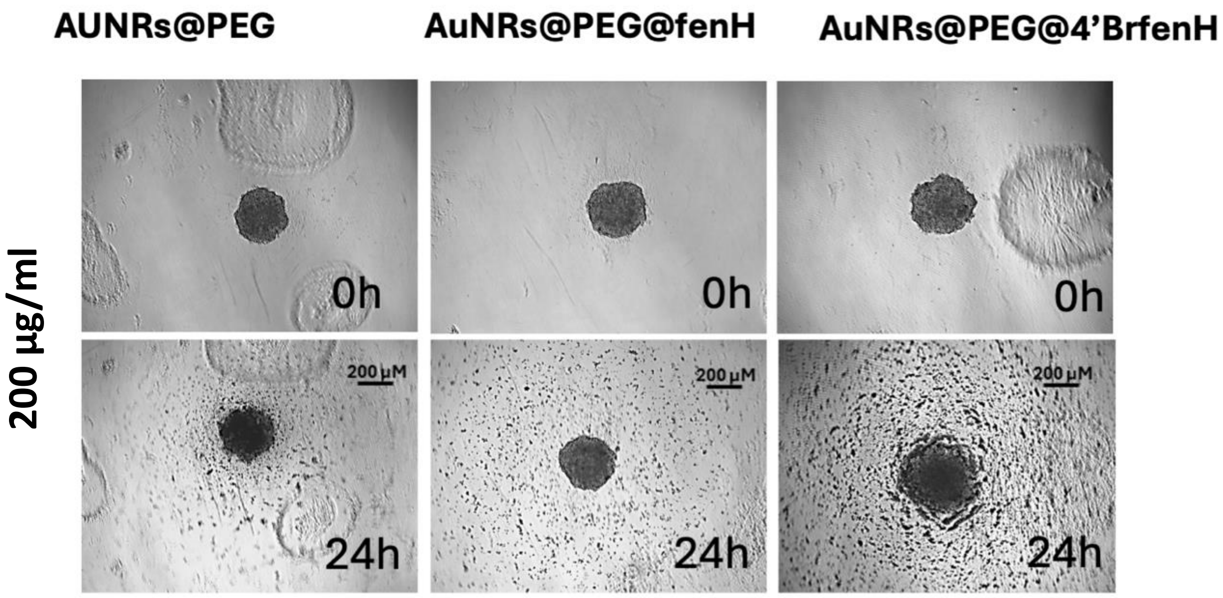
AUNRs@PEG
AuNRs@PEG@fenH
AuNRs@PEG@4'BrfenH
200 μg/ml
0h
0h
0h
200 μM
200 μM
200 μM
24h
24h
24h

Figure S11. Cell viability of MCF-7 in 2D cell cultures in the presence of AuNRs@PEG, AuNRs@PEG@fenH and AuNRs@PEG@4'BrfenH at 1, 50 and 200 µg/mL. Representative images of MCF-7 after 0 and 24 h of treatment.

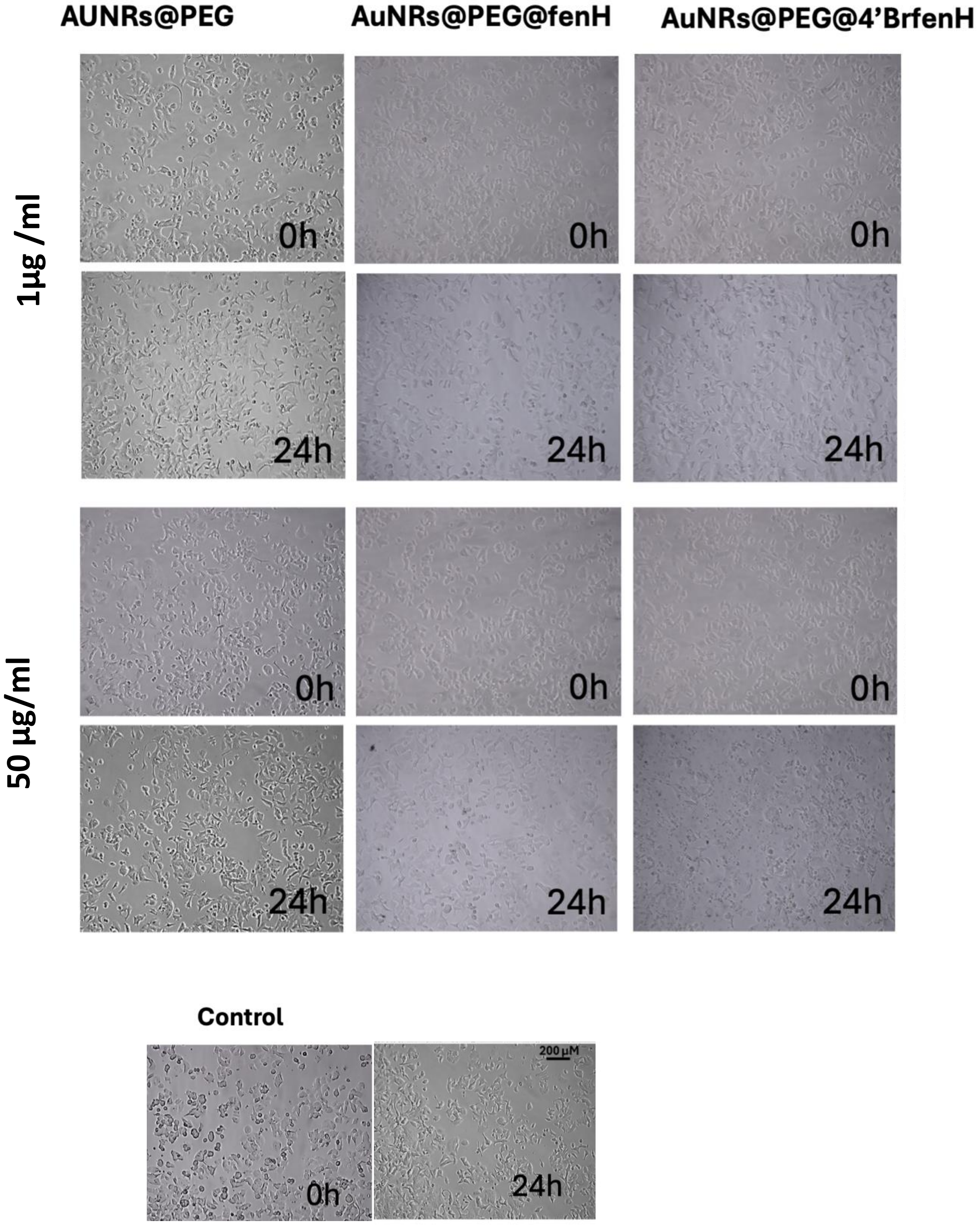

(continued)

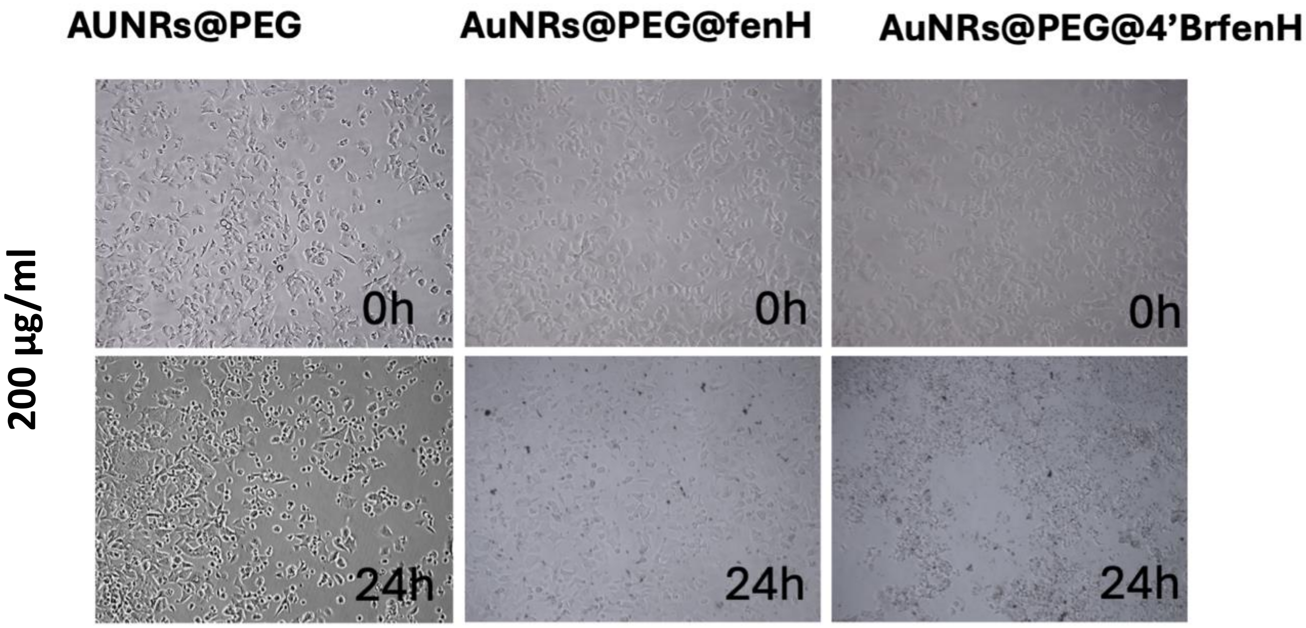
AUNRs@PEG
AuNRs@PEG@fenH
AuNRs@PEG@4’BrfenH
200 µg/ml
0h
0h
0h
24h
24h
24h

Figure S12. Spheroids compactness of MCF-7 in 3D cultures. Representative images of MCF-7 spheroids in the presence of AuNRs@PEG, AuNRs@PEG@fenH and AuNRs@PEG@4’BrfenH at 1, 50 and 200 µg/mL after 0 and 24 h of treatment.

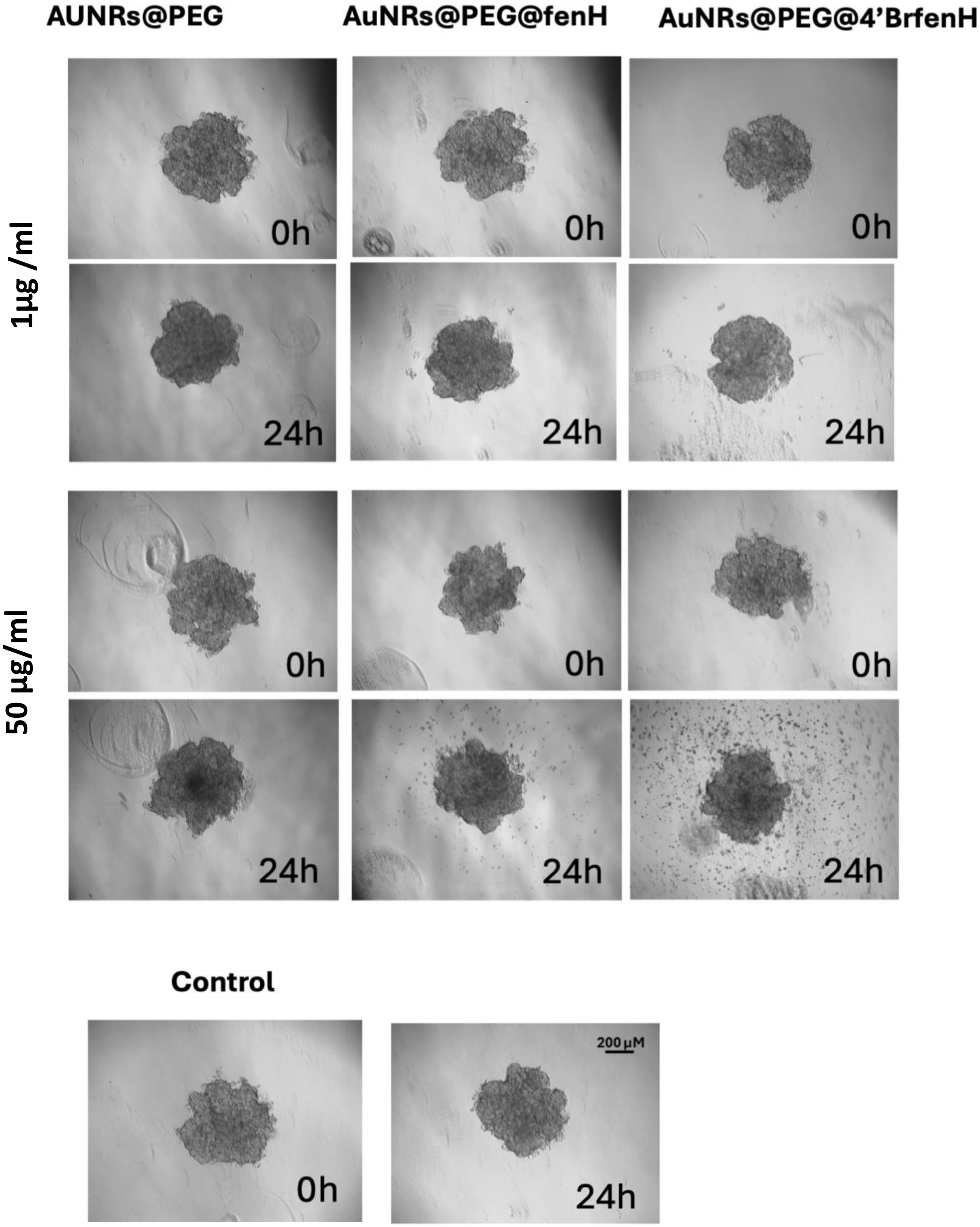

(continued)

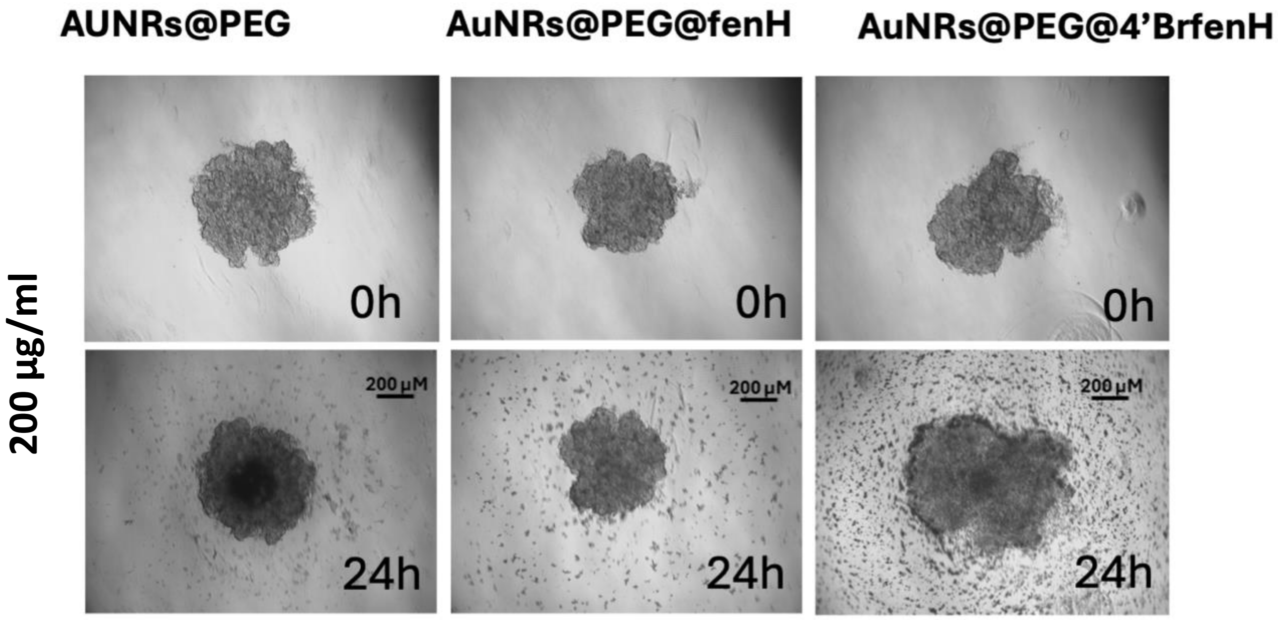
AUNRs@PEG
AuNRs@PEG@fenH
AuNRs@PEG@4'BrfenH
200 µg/ml
0h
0h
0h
200 µM
200 µM
200 µM
24h
24h
24h
